# Ancient Haplotypes at the 15q24.2 Microdeletion Region Are Linked to Brain Expression of *MAN2C1* and Children's Intelligence

**DOI:** 10.1371/journal.pone.0157739

**Published:** 2016-06-29

**Authors:** Alejandro Cáceres, Tõnu Esko, Irene Pappa, Armand Gutiérrez, Maria-Jose Lopez-Espinosa, Sabrina Llop, Mariona Bustamante, Henning Tiemeier, Andres Metspalu, Peter K. Joshi, James F. Wilsonx, Judith Reina-Castillón, Jean Shin, Zdenka Pausova, Tomáš Paus, Jordi Sunyer, Luis A. Pérez-Jurado, Juan R. González

**Affiliations:** 1 ISGlobal, Center for Research in Environmental Epidemiology (CREAL), Barcelona, Spain; 2 Universitat Pompeu Fabra, Barcelona, Spain; 3 Centro de Investigación Biomédica en Red en Epidemiología y Salud Pública (CIBERESP), Madrid, Spain; 4 Department of Genetics, Harvard Medical School, Boston, Massachusetts, United States of America; 5 Broad Institute, Cambridge, Massachusetts, United States of America; 6 Division of Endocrinology, Children’s Hospital Boston, Boston, Massachusetts, United States of America; 7 Estonian Genome Center, University of Tartu, Tartu, Estonia; 8 School of Pedagogical and Educational Sciences, Erasmus University Rotterdam, Rotterdam, The Netherlands; 9 Generation R Study Group, Erasmus Medical Center, Rotterdam, The Netherlands; 10 Department of Experimental and Health Sciences, Universitat Pompeu Fabra, Barcelona, Spain; 11 Centro de Investigación Biomédica en Red de Enfermedades Raras (CIBERER), Madrid, Spain; 12 Hospital del Mar Research Institute (IMIM), Barcelona, Spain; 13 Epidemiology and Environmental Health Joint Research Unit, FISABIO–Universitat Jaume I–Universitat de València, Valencia, Spain; 14 Genomics and Disease Group, Centre for Genomic Regulation (CRG), Barcelona, Spain; 15 Department of Child and Adolescent Psychiatry/Psychology, Erasmus University Medical Center-Sophia Children’s Hospital, Rotterdam, The Netherlands; 16 Department of Psychiatry, Erasmus Medical Center, Rotterdam, The Netherlands; 17 Institute of Molecular and Cell Biology, University of Tartu, Tartu, Estonia; 18 Centre for Global Health Research, Usher Institute for Population Health Sciences and Informatics, University of Edinburgh, Edinburgh, Scotland; 19 MRC Human Genetics Unit, Institute of Genetics and Molecular Medicine, University of Edinburgh, Edinburgh, Scotland; 20 Hospital for Sick Children, University of Toronto, Toronto, Ontario, Canada; 21 Rotman Research Institute, University of Toronto, Toronto, Ontario, Canada; 22 Department of Mathematics, Universitat Autònoma de Barcelona, Bellaterra (Barcelona), Spain; Hospital Israelita Albert Einstein, BRAZIL

## Abstract

The chromosome bands 15q24.1-15q24.3 contain a complex region with numerous segmental duplications that predispose to regional microduplications and microdeletions, both of which have been linked to intellectual disability, speech delay and autistic features. The region may also harbour common inversion polymorphisms whose functional and phenotypic manifestations are unknown. Using single nucleotide polymorphism (SNP) data, we detected four large contiguous haplotype-genotypes at 15q24 with Mendelian inheritance in 2,562 trios, African origin, high population stratification and reduced recombination rates. Although the haplotype-genotypes have been most likely generated by decreased or absent recombination among them, we could not confirm that they were the product of inversion polymorphisms in the region. One of the blocks was composed of three haplotype-genotypes (N1a, N1b and N2), which significantly correlated with intelligence quotient (IQ) in 2,735 children of European ancestry from three independent population cohorts. Homozygosity for N2 was associated with lower verbal IQ (2.4-point loss, p-value = 0.01), while homozygosity for N1b was associated with 3.2-point loss in non-verbal IQ (p-value = 0.0006). The three alleles strongly correlated with expression levels of *MAN2C1* and *SNUPN* in blood and brain. Homozygosity for N2 correlated with over-expression of *MAN2C1* over many brain areas but the occipital cortex where N1b homozygous highly under-expressed. Our population-based analyses suggest that *MAN2C1* may contribute to the verbal difficulties observed in microduplications and to the intellectual disability of microdeletion syndromes, whose characteristic dosage increment and removal may affect different brain areas.

## Introduction

The chromosome bands 15q24.1-15q24.3 harbour a complex genomic region with multiple large blocks of segmental duplications (A through E) that mediate recurrent rearrangements, including inversions, deletions and duplications of variable size and extent [1–3]. Both, microdeletions and microduplications of this region cause unusual facial morphology along with intellectual disability, speech delay and autistic features [4–7]. Most reported deletions associated with phenotype include the 1.1 Mb critical region located between blocks B and C and also the 0.6 Mb C–D region where smaller deletions have been found in at least two patients with borderline intellectual disability [4]. Thus, while the severe core cognitive deficits of the 15q24 microdeletion syndrome are thought to be due to deletion of genes between B and C, some of the genes located between blocks C and D must also be important for normal development and behaviour.

The finding of inversion polymorphisms in the region suggests suppression of recombination and possible support for extended haplotypes [2]. It has been previously shown that the imprints of inversions can result in extended regions with high linkage disequilibrium (LD) and points where there is higher linkage at distant points than at the immediate neighbourhood [8–10]. The convergence of these two features has been used to infer inversion status in other inversions [11]. Here we first investigated the haplotype structure of 15q24 and its potential link to previously reported inversions. We have found four contiguous extended haplotype blocks, detectable by differences in linkage disequilibrium (LD) between SNP blocks and haplotype divergence clades [11–12]. Given the reported implication of gene dosage effects of this region in autism and cognitive deficits, we aimed to investigate the evolutionary history, the effect on gene expression and putative influence of the haplotype-genotypes on autism susceptibility and on intelligence quotient (IQ) of children and adolescents recruited from the general population. These population-based analyses were used to determine the genes that might also contribute to the cognitive impairments of the microdeletion and microduplication syndromes.

## Results

### Characterization of haplotypes blocks at 15q24.2

**Fig 1:**
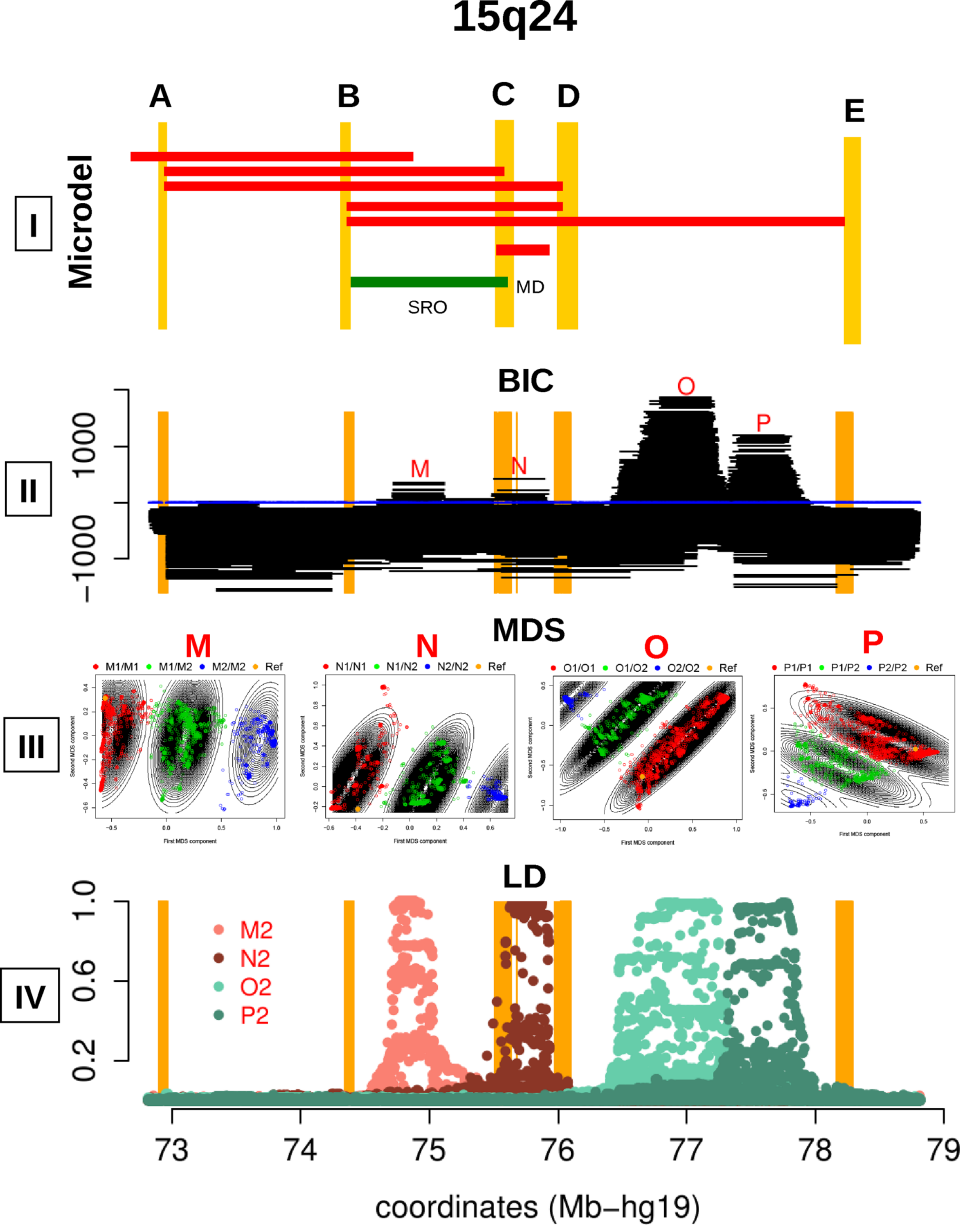
Long haplotype structures in 15q24. **I)** Genomic location of the region between 15q24.1-15q24.3 prone to microdeletion and micro duplication syndromes. Segmental duplication blocks A to E are indicated in yellow. Red blocks show the microdeletion cases, adapted from Mefford et al. [4]. The red block marked with MD is a case with the minimum deletion segment (MD in the figure). Also, the green block illustrates the inversion discussed by Antonacci et al. [2] coinciding with the smallest region of overlap (SRO) identified by Magoulas and El-Hattab [3]. **II)** inveRsion scan over the region. Clear signals of LD differences between SNP blocks are detectable and marked with M, N, O, P. **III)** Haplotype-genotype clustering of Multidimensional (MDS) Analysis by invClust within segments M, N, O and P. **IV)** Blocks of LD (r^2^) between SNPs in the regions M, N, O and P and the haplotype-genotype calls made with invClust.

We used two recent bioinformatics methods, inveRsion and invClust within 15q24.1-15q24.3 (Fig 1I), to detect linkage disequilibrium differences and extended haplotype-genotype structures that could be caused by suppressed recombination and even linked with inversion polymorphisms, see more details in the Methods section. Scanning with inveRsion SNP data, of 505 European subjects from the 1000 genomes project, the region between 72–79 Mb of chromosome 15 (hg19), we detected four positive signals (BIC>0) of LD differences (Fig 1II). The first signal (M) is within the previously reported 1.1Mb inversion between segmental duplications B and C and extends between: 74.71–75.12Mb (hg19) [1–2]. The second block (N) with significant LD differences corresponded to a 0.4 Mb interval between 75.5–75.9Mb, within the segmental duplications C and D. The other two signals (O and P) were obtained between 76.46–77.31Mb and 77.3–77.87Mb, within duplications D-E.

We then used the multivariate method, invClust, to determine the haplotype-genotypes of the M, N, O and P regions (Fig 1III). We clustered the first two MDS components into genotypes where the reference genome was mapped to the non-variant alleles M1, N1, O1 and P1, and we denoted the variant haplotypes M2, N2, O2 and P2. We found very low LD between all haplotype-genotypes (r^2^<0.06). We determined the extent of the haplotypes-genotypes by computing the LD (r^2^) between the haplotype-genotypes and the SNPs within the 15q24 region (Fig 1IV). To confirm the allelic structure of the haplotypes we tested their Mendelian inheritance in 2,259 European and 303 non-European trios from the Autistic Genome Project (AGP). We observed a very low rate in transmission error 0.4% for M1/M2, 0.6% for N1/N2, 1.1% for O1/O2 and 1.9% for P1/P2.

**Fig 2:**
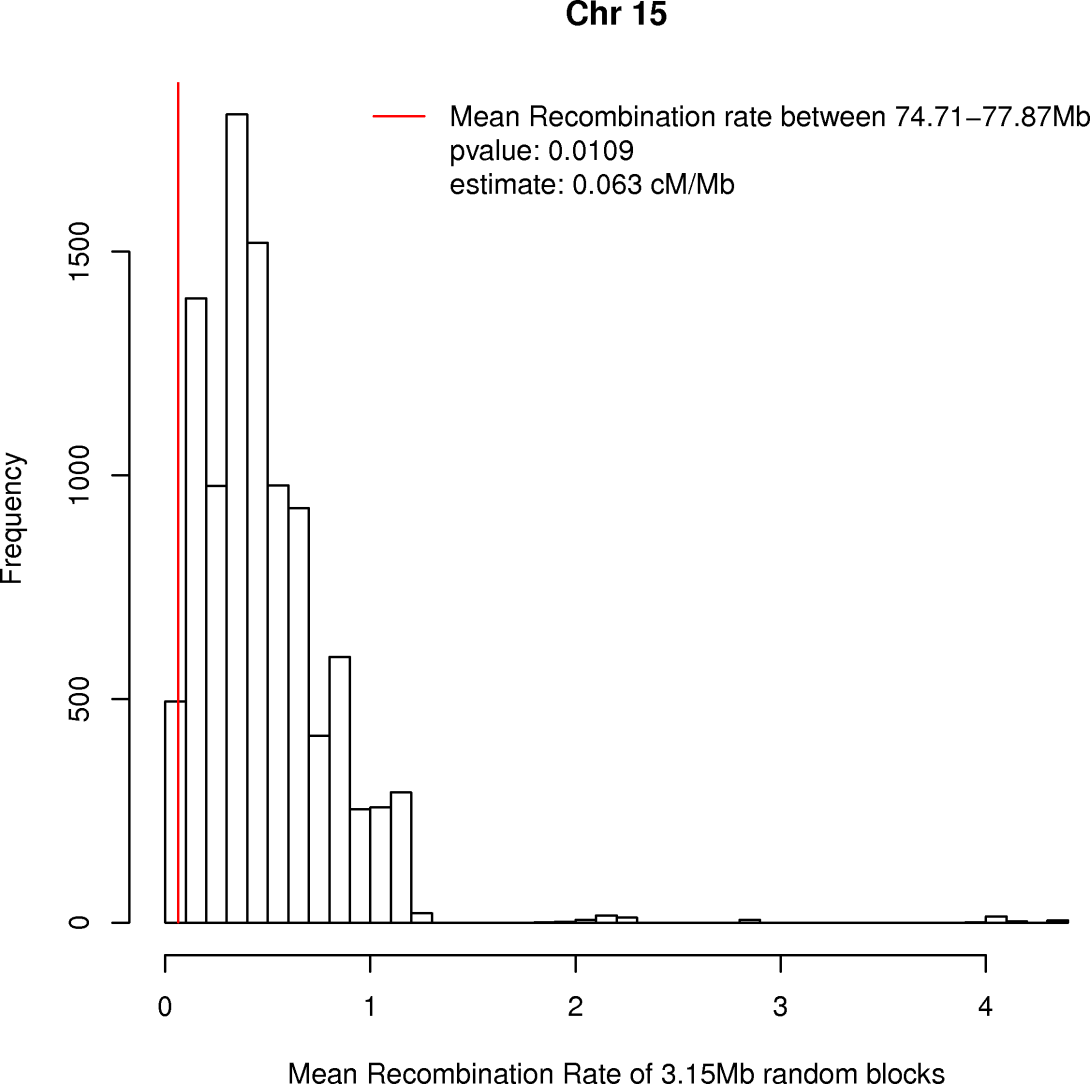
Recombination rate in the region extending the four haplotype blocks M, N, O and P in 15q24. The figure shows significant reduction of the in the recombination rate of the 3.15Mb segment between 74.71–77.87Mb, as compared with the mean recombination of 10,000 random segments of similar length from chromosome 15.

We analysed the recombination rate from HapMap II (version 2011–01, http://hapmap.ncbi.nlm.nih.gov/downloads/recombination/) data of the entire 3.15 Mb region comprising the four haplotype blocks (74.71–77.87Mb, hg19). We randomly selected 10,000 segments of similar size over chromosome 15 and computed their mean recombination rate. We then compared the mean recombination rate of the M-P block (0.063 cM/Mb) with the distribution of values obtained in the random selection and found that this segment has a significant reduction in recombination rate (p-value = 0.01); see Fig 2.

**Fig 3:**
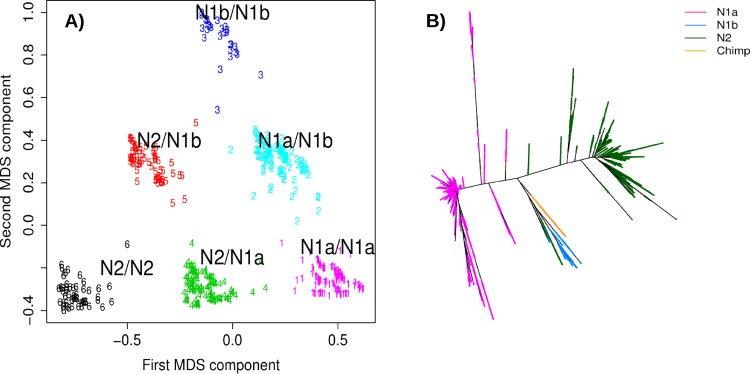
Haplotype structure of three clades in the N block. **A)** The MDS analysis in the CD region reveals three clusters that correspond to six different genotypes, with low Mendelian errors of transmission. **B)** NJ plot showing the phylogenetic relationships between N1a, N1b and N2. The chimp allele is found in the clade N1b.

We further observed that within the N block subjects could be classified into six clusters consistent with the haplotype-genotypes of two other possible clades, in addition to N2 (Fig 3A). In the MDS analysis within the C-D block, we found that each cluster could represent six possible haplotype-genotypes of three different haplotype groups, namely N2, N1a and N1b. The three extreme clusters would represent the homozygous individuals (N1a/N1a: cluster 1, N1b/N1b: cluster 3, N2/N2: cluster 6) and the clusters in between two homozygous groups would contain the corresponding heterozygous individuals (N1a/N1b: cluster 2, N1a/N2: cluster 4, N1b/N2: cluster 5). We confirmed the allelic structure of the haplotypes as there were no Mendelian errors in the 60 HapMap CEU and YRI trios and very low errors in the 2,259 AGP trios for the three predicted haplotypes: 0.4% for Europeans and 1.9% for non-Europeans (S1 File). Thus, the haplotype-genotyping was highly accurate for inferences in large population datasets.

We computed LD r^2^ between the SNPs in the 15q24.2 region and the haplotype status (N1a, N1b, N2) of CEU individuals in the 1000 Genome data. We found over 250 SNPs that could be used to tag a given haplotype with r^2^>0.9; see S2 File were the LD for YRI and CHB-JPT are also reported. Phylogeny analysis for N1a, N1b and N2 homozygous, on the 26 populations of the 1000 Genomes, showed clades that follow each haplotype group rather than population membership, suggesting greater genetic variability between the haplotype groups than between ancestral populations (Fig 3B). We observed that the chimp sequence mapped to N1b; the less frequent of the three haplotypes in Europeans: N2: 59%, N1a: 24%, N1b: 17%.

### Population frequency of haplotype blocks at 15q24

**Fig 4:**
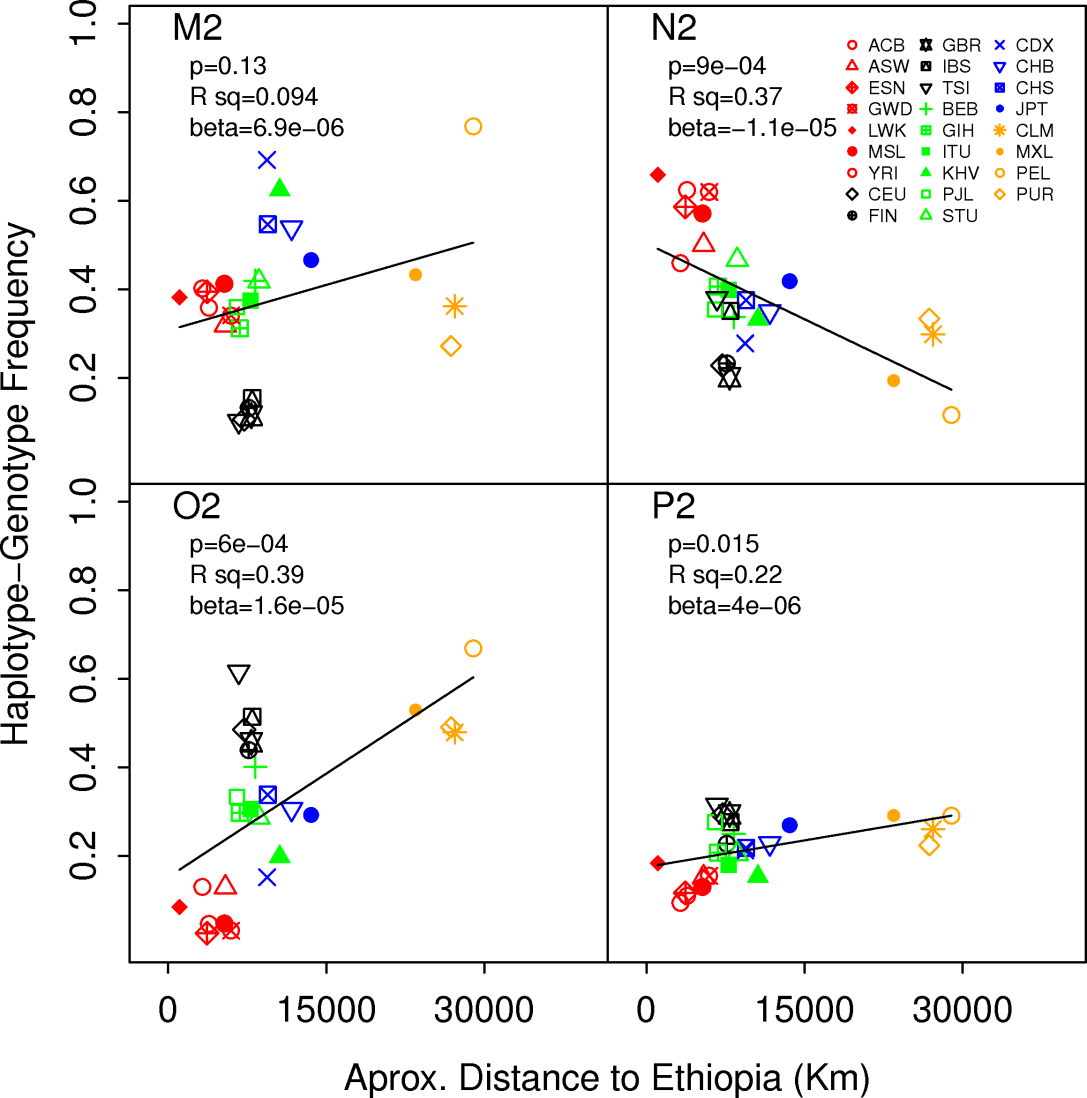
Global distribution of M2, N2, O2 and P2 haplotypes in 1000 Genomes populations. Haplotypes frequencies strongly correlated with distance of the distance from Ethiopia.

We analysed all 26 populations from the 1000 Genomes project to determine the global frequency of all four M2, N2, O2 and P2 haplotype groups. We observed that all haplotype-genotypes were in Hardy Weinberg Equilibrium (S1 Table), with the only exception of P2 for the KHV and BEB populations (pvalue < 0.01). We found that the four frequencies for M2, N2, O2 and P2 are highly stratified by population origin and follow a clinal distribution consistent with an out of Africa expansion (Fig 4). We tested whether the distance from Ethiopia could explain more the differences between the population frequencies than genetic drift alone. For each haplotype block, random samples of 10,000 thousand SNPs across the genome were drawn with similar mean frequencies in Africa. For each SNP, we computed the explained variance given by the R^2^ value obtained from the regression model between the population frequency and the distance from Ethiopia. The R^2^ for the haplotypes M2, N2, O2 and P2 was compared with the null distributions obtained from the sampling. We thus observed that the distance from Ethiopia explains more the differences between population frequencies of O2 (p-value = 0.02) than what would be expected from genetic drift alone (S1 Fig). The N2 allele had a tendency to significance (p-value = 0.06). We also observed that two SNPs tagging N2 and O2, rs4462560 and rs9635320, respectively, showed significant signals of selection by iHS measures (|iHS|>2) in YRI (iHS in rs4462560 = 3.008 and iHS in rs9635320 = -3.822) as reported in the haplotter database (http://haplotter.uchicago.edu/instruction.html) [13]. We could not eliminate genetic drift as a potential driver of heterozygosities of M2 and P2 haplotypes nor in the F_ST_ values in any of the four haplotypes, which nonetheless increased with distance from Ethiopia, as expected (S2 Fig).

### Haplotypes and structural variants

We compared the entire ~7 Mb human genomic region at 15q24 (72–79 Mb, hg19) with the orthologous region in the rat genome (genome assembly rn5.0) to establish the blocks of the synteny, using ArkMAP [14]. We observed a complex evolutionary history of the region with several inversion and transposition events with some breaks of synteny at the regions harbouring the blocks segmental duplications in the human genome (S3 Fig). We found a linage specific inversion in *Homo sapiens* for the region D-E with respect to C-D and a deletion between the regions B-C and C-D. In addition, an additional block flanked by segmental duplications and distal to D-E has been translocated and the generation of the related segmental duplication blocks A-D suggest additional evolutionary rearrangements not fully resolved. A similar pattern to the rat alignment was observed when human was aligned to the mouse genome; however, no such brakes were observed with respect to the orangutan or macaque (S4 Fig). A detailed look into BAC libraries for both species revealed, however, evolutionary inversions and rearrangements in macaque across the orthologs to the human segmental duplications.

We investigated the extent to which the haplotype structure can be linked with reported polymorphic inversions in the region. Four individuals (NA18555, NA12156, NA19129 and NA19240) from 1000 Genomes have been reported with an inversion polymorphism between B-C region [1–2]. We found that three of these individuals had genotypes M1/M2, and one was M1/M1, suggesting M2 could be supported by the inversion polymorphism. However, out of the 29 individuals from the 1000 Genomes reported as non-inverted homozygous, we found 15 M1/M1, 8 M1/M2 and 6 M2/M2. The substantial disagreement between M1 and non-invariant status suggests an occurrence of the inversion in an older M2 background or recurrent unrelated events, and ruling out the reported inversion as a possible cause for the haplotype structure at M.

We searched in three BAC libraries and found that, in accordance to the top tag SNPs for N2, N1a and N1b, libraries RP11, CTD and CTA most likely correspond to haplotypes N2, N1a and N1b, respectively (S3 File). We then observed that there are different gaps in each of the libraries: 1) library RP11 has a gap in the segmental duplication D and in a point between C and D; 2) library CTD does not cross segmental duplications B nor C; and 3) library CTA does not cross either C or E (S4 Fig). The gaps in library CTD (N1a) are consistent with reported inversions at the proximal region B-C. Interestingly, all four reported individuals with inversions at B-C are heterozygous for N1a at C-D; NA18555 and NA19129 are N1a/N2 and NA12156 and NA19240 are N1a/N1b, suggesting that the inversion in B-C could have occurred in the long haplotype M2-N1a. However, we also observed that no inversion can be present between blocks C and D in any of the haplotypes. This could not be confirmed by interphase FISH where the BACs within C-D are too close to produce images with appropriate resolution (S5 Fig). However, interphase FISH had enough resolution to discard inversions in intervals B-C or B-D in individuals with haplotype-genotypes N2/N2 (NA19146), N2/N1a (NA19213) and N2/N1b (NA19189), see S6 Fig.

### Association of haplotypes-genotypes at 15q24.2 with IQ

As copy number changes in the region have been implicated in autism and intellectual disability, we first tested the transmission disequilibrium from heterozygous parents to autistic children for the haplotypes M2, N2, O2 and P2. We performed a transmission disequilibrium test (TDT) on the 2,259 European and 303 non-European trios of AGP. We did not find any significant association for any of the haplotypes (M2: TDT-χ^2^ = 0.23, p-value = 0.62; N2: TDT-χ^2^ = 0.09, p-value = 0.76, O2: TDT-χ^2^ = 1.63, p-value = 0.20, P2: TDT-χ^2^ = 0.24, p-value = 0.62).

We then inferred the M, N, O and P haplotypes in 909 Spanish children in the INMA (INfancia y Medio Ambiente) cohort and tested their association with IQ. For haplotypes at M, O and P, we used additive genetic models whereas for the three haplotypes at N, we used recessive models for N2, N1a and N1b (Figure A in S7 Fig), to assess the specific contribution of each haplotype to the verbal and non-verbal IQ. We fitted Gaussian regression models for the normalized IQ measurements on all genetic models and adjusted for sex, age at test administration and first two genome-wide PCA components; see Table 1. We found a significant association between N2 and 2.9-point reduction in verbal-IQ.

**Table 1:** Associations between IQ components and haplotypes M, N, O and P in the 909 Spanish children from the INMA cohort.

	Verbal IQ		Non-Verbal IQ	
	Estimate	P-value	Estimate	P-value
**M2**	0.68	0.39	0.24	0.66
**N2-rec**	-2.93	**0.025**	-1.20	0.18
**N1a-rec**	0.40	0.67	0.99	0.13
**N1b-rec**	0.19	0.91	-2.30	0.070
**O2**	-0.29	0.63	0.26	0.52
**P2**	-0.56	0.35	-0.76	0.067

Additive models were fitted for M2, O2 and P2 while recessive models were fitted for the three haplotypes at N (N2, N1a and N1b). Homozygocity for N2 is significantly associated with reductions in verbal-IQ.

We then aimed to replicate the association of haplotype-genotypes at N in the Dutch GenR (Generation R) and Canadian SYS (Saguenay Youth Study) cohorts. As in the previous analysis, in the 1,236 children from GenR population cohort, we identified six clusters, corresponding to the genotypes of three possible haplotypes in the region (Figure B in S7 Fig). Clusters were numbered according with the tag SNPs in S2 File. For the SYS cohort of adolescents, we had more individuals genotyped (children and parents) but lower density of SNPs. Because only 8 SNPs from S2 File were available for the analysis, we could only use the first MDS component where we found a clear 5-cluster pattern (Figure C in S7 Fig). The N1b homozygous were inferred as those individuals who are simultaneously non-variant homozygous for N1a and N2. To assess the accuracy of this inference, we used the same eight SNPs in the European individuals of the 1000 Genomes and follow a similar procedure. We found that N2 could be inferred with 100% accuracy, while N1a and N1b were inferred with 99.4% and 99.6% accuracies, respectively.

**Fig 5:**
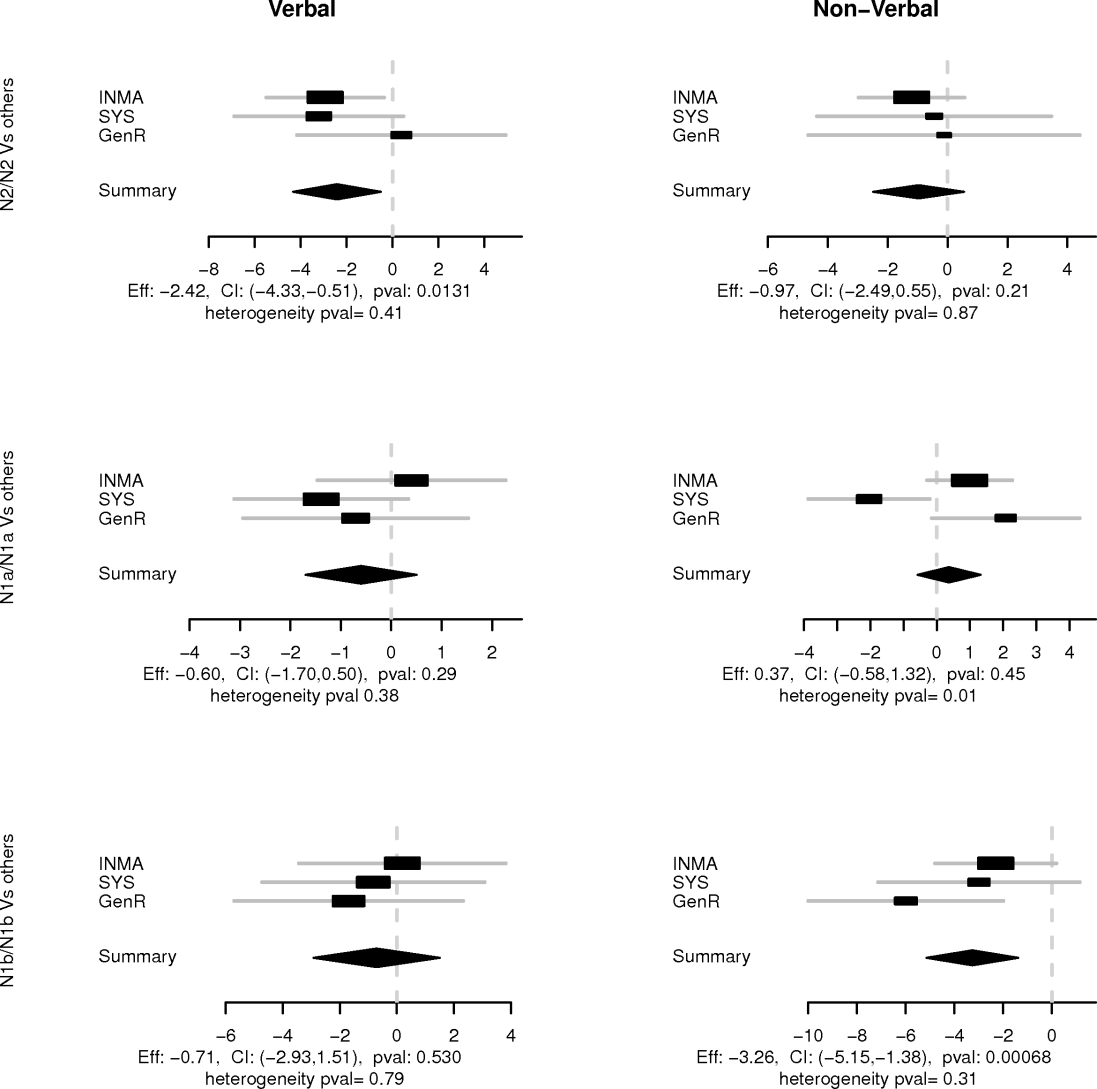
Association between IQ and homozygosity for N2, N1a and N1b. The figure shows the meta-analysis of three studies (INMA, SYS and GenR) for the association between IQ measures and homozygosity for the N2, N1a and N1b alleles.

We performed a meta-analysis where the weights were the reciprocal of the estimated variance (Fig 5), fixed effects were considered to account for between cohort variability and no significant heterogeneity between cohorts was observed in all significant results. We found the N1b allele correlated with a 3.2-point loss in non-verbal intelligence (p-value = 0.0006). In addition, homozygosity for the N2 haplotype was the only genetic model that correlated with verbal IQ (mean decrement of 2.4 points, p-value = 0.01). As expected, for all three haplotype tests, we found high correlation between verbal and non-verbal IQ estimates (cor = 0.81 for N2, cor = 0.70 for N1a, and cor = 0.98 for N1b).

We analysed the 2,215 Scottish adults of ORCADES to investigate if the association in verbal IQ is also present in adults. For this specific cohort, we did not find a significant association (N2: p-value = 0.2, N1a: p-value = 0.5, N1b: p-value: 0.06). However, since the association with N2 homozygosity was also negative and comparable to that observed in children, we found an increment on the statistical significance in the overall meta-analysis (2.0-point loss, p-value = 0.007); S8 Fig.

### Functional correlation of N2 alleles with gene expression

**Fig 6:**
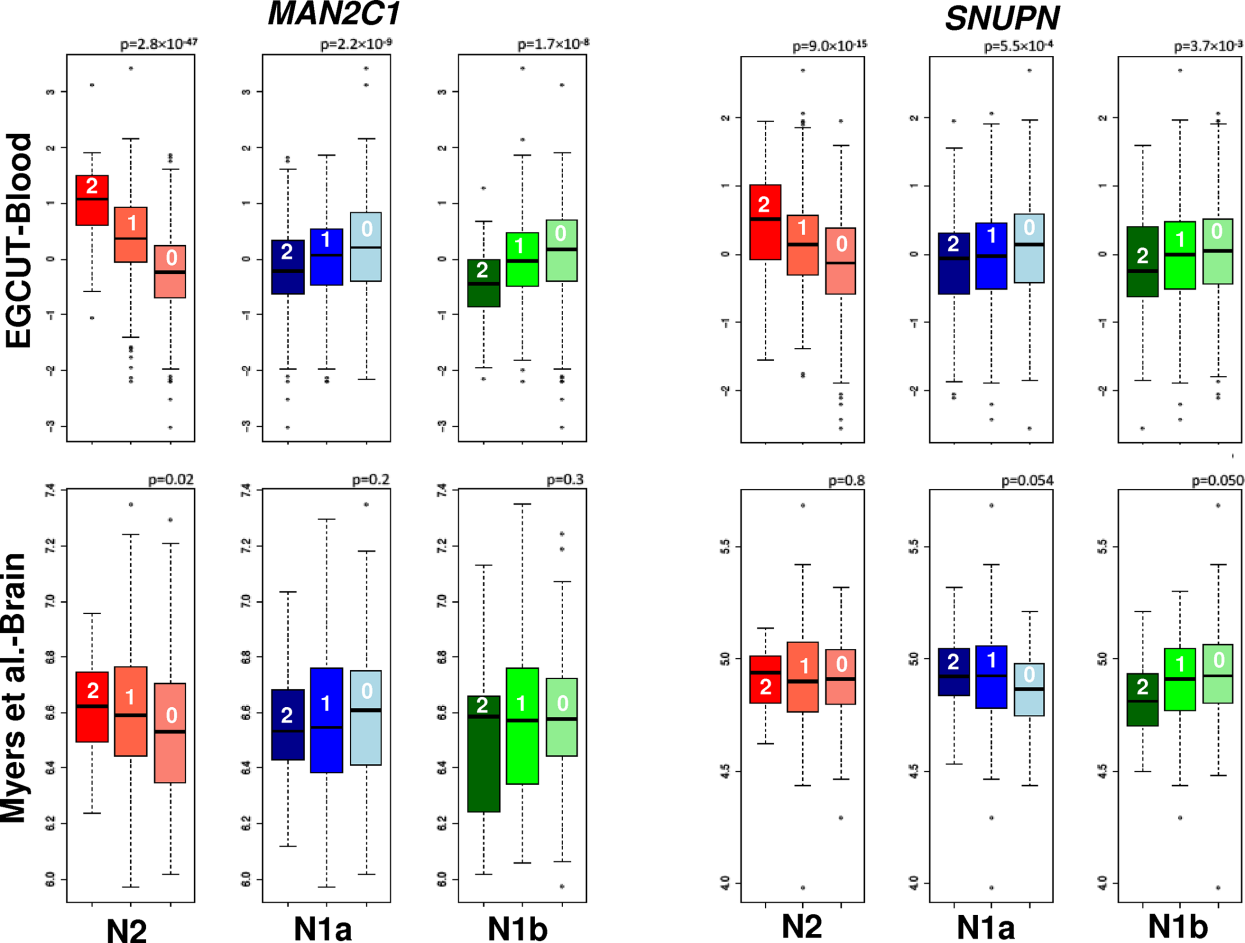
Association between N2, N1a and N1b alleles and the gene expressions of *MAN2C1* and *SNUPN* in blood and brain. Logarithm of the expression of *MAN2C1* and *SNUPN* in the peripheral blood of 882 Estonians (EGCUT) and 193 deceased un-demented individuals (Myers et al. [33]) as function of the genotype-haplotypes. Numbers in the box-plots correspond to the number of haplotypes. The expression *MAN2C1* and *SNUPN* strongly correlated with the three haplotype-genotypes in blood and was validated for N2 (*MAN2C1*) and N1b (*SNUPN*) in brain.

We performed association tests between the normalized gene expression within the C-D region in 15q24.2 and the haplotypes-genotypes at N. We first analysed expression data of the 882 Estonians from the EGCUT study for each specific allele and found significant associations of local genes (S2A Table). The expression of *MAN2C1* increased per N2 haplotype (p-value<10^−46^), and decreased with both N1a (p<10^−8^) and N1b (p<10^−7^) haplotype (Fig 6). An additional significant association was found for *SNUPN*, which followed the same pattern of *MAN2C1* (N2: p-value <10^−15^, N1a: p-value = 0.0005, N1b, p-value = 0.003). Using transcriptomic data in lymphoblastoid cell lines of the 105 CEU individuals of HapMap, we validated the expression pattern of *MAN2C1* with respect to the N2 (p-value<10^−4^) and N1a (p-value<10^−5^) haplotypes (S2B Table). We then used the brain expression data of 193 control individuals. In agreement with the previous analyses, we found that the N2 haplotype was associated with higher *MAN2C1* expression in cerebral cortex (p-value = 0.02), see Fig 6. However, we did not find significant associations with N1a and N1b. We also tested associations between the haplotype-genotypes and the expression of *SNUPN* in brain and validated a significant reduction per N1b allele (p-value = 0.05), see Fig 6.

**Fig 7:**
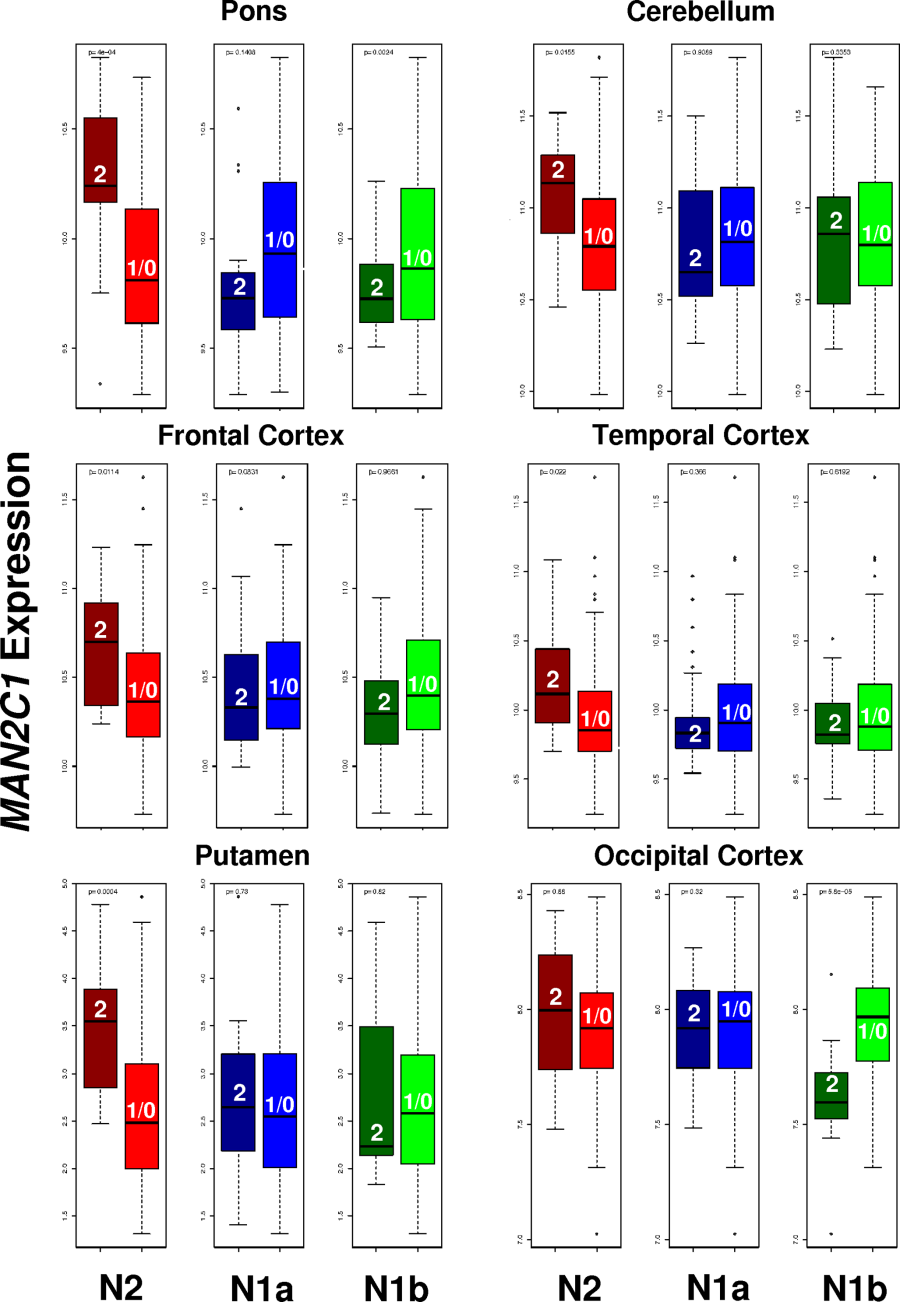
Significant deregulation of *MAN2C1* expression in the brain for N2, N1a and N1b homozygous. Numbers in boxplots correspond to the number of haplotypes considered in each group. *MAN2C1* is ubiquitously over-expressed for N2 homozygous in brain, including pons, cerebellum, frontal cortex, temporal cortex and putamen, but not in occipital cortex, where N1b homozygous are strongly associated with the under-expression of the gene. Significant results in pons, cerebellum, frontal cortex, temporal cortex were obtained from the BRAINeQTL study while those for putamen and occipital cortex where obtained from the BRAINEAC project. Other brain regions in both data-sets showed non-significant associations.

We analysed expression data from the BRAINeQTL study and BRAINEAC project to investigate the regional difference of gene expression in brain, see Fig 7. For the BRAINeQTL study, we correlated the expression of *MAN2C1* in four different brain areas for 148 subjects and tested recessive models for the haplotypes to help interpret previous IQ correlations. We found that the N2 allele is associated with increments of *MAN2C1* transcription in pons (p-value = 0.0004), cerebellum (p-value = 0.01), frontal cortex (p-value = 0.01) and temporal cortex (p-value = 0.02) while homozygous for N1b had significant decrements of gene expression only in pons (p-value = 0.002) and no significant association was found for the N1a allele. In the BRAINEAC data-set of 134 individuals, we selected 36 intragenic probes within *MAN2C1* and tested the correlation between the recessive models for each allele and the expression of the gene across 10 different brain regions. We found only two probes that survived Bonferroni correction within each region for *MAN2C1*. The first one was in the putamen for N2 homozygosity (p-value = 0.0004). The second probe confirmed the high correlation in the occipital cortex only with N1b (p-value<10^−4^) and not for N2 (p-value = 0.8). We did not find significant results in the other areas of the brain.

As correlations of gene expression were performed for adult brain, we further enquiry the Human Brain Transcriptome database (http://hbatlas.org/) to determine whether *MAN2C1* expression changed during brain development. S9 Fig shows a clear continuous growth of the gene's expression in brain throughout human life, starting prenatally, supporting an important role of *MAN2C1* in neurodevelopment.

## Discussion

We revealed the existence of a complex haplotype structure at the microdeletion region 15q24. Four large blocks of haplotype-genotypes were delimited by segmental duplications and showed strong stratification in global populations. These ancestral haplotypes of the region have been likely generated by suppression of recombination and two of them show apparent selection signals. One of these blocks is between segmental duplications C-D. We found that the block’s three-haplotype structure (N2, N1a and N1b) correlated with IQ in children and with up-regulation and down-regulation of local of genes, *MAN2C1* and *SNUPN*, which depended on brain region.

Microduplications involving C-D have been associated with autistic features and language problems while their haploinsufficiency has been related to intellectual disability [4]. Such gene dosage associations fit well with our observations in normal developing population showing that 1) higher expression of *MAN2C1* associated to the N2 haplotype is linked with lower verbal IQ, and 2) lower expression of *MAN2C1* related to the N1b haplotype is linked to reductions on non-verbal IQ. Remarkably, up-regulation of *MAN2C1* by N2 is stronger in the frontal and temporal cortex, where language and high cognitive functions are processed, while down-regulation by N1b is most prominent in the occipital cortex, where it could affect processing of visual stimuli and thus influence non-verbal IQ. Our findings therefore suggest that *MAN2C1* can be an important contributor to the cognitive impairments of both microdeletions and microduplications.

Given that the 15q24.2 haplotypes comprise a region in which copy number changes are causative of autism and cognitive impairment [4], we hypothesized that haplotype-genotypes could be susceptibility factors for autism and/or could be linked to cognitive variability in children. We did not find significant associations with autism but observed relevant yet weak correlations between the haplotypes and IQ profiles. The association in the meta-analysis for verbal IQ is improved with the inclusion of a large adult cohort, suggesting that the genetic effect may still be relevant later in life. In this study, similarly to large epidemiological studies that combined MSCA and WISC tests [15], we assumed that diverse IQ measures can be meta-analysed. While this constitutes a general drawback in neuro-epidemiological studies, the expectation of combining different correlated measurements is to reduce statistical power rather than increment false positives. A previous GWAS, based in the meta-analysis of different IQ measurements on 18,000 children (including SON-R 2 ½ -7 and WISC amongst many others), did not find any significant SNP at genome-wide significance and only one finding was reported at a gene-set level (*FNBP1L*, uncorrected p-value = 0.003) [16]. In particular, none of the SNPs or genes in the 15q24 region was found significant in that study. In a genome-wide context, our findings could be similarly under-power by the variability of the IQ measures and age ranges. However, our study is akin a “candidate gene” study and require no correction for multiple comparisons and, as such, we do find statistically significant results. In addition, an important aspect of our approach was to identify the genes that may be causative of the cognitive impairments in genetic syndromes through population based analyses. We thus observed that *MAN2C1* and *SNUPN* are the most likely genes whose variability can affect cognition through gene dosage.

Out of the 10 single copy genes located within the C-D interval, the expressions of *SNUPN* and *MAN2C1* were consistently up-regulated at the N2 allele and down-regulated at the N1b configuration, both in blood and brain tissues. *SNUPN* encodes snurportin 1, a protein required, trough interaction with the spinal muscular atrophy protein *SMN*, for the nuclear import of snRNPs and splicing regulation of multiple genes [17]. As such, *SNUPN* de-regulation can affect the central nervous system but there is not yet evidence for a more direct relation to cognition.

*MAN2C1* has been shown to have a dual function. *MAN2C1* encodes the alpha-mannosidase, class 2C, member 1 that has been shown to regulate protein N-glycosylation and apoptosis. The N-Glycoproteome maps mainly to blood but the highest amount of organ specific N-glycosylation sites in mice has been observed in the brain [18]. As the *MAN2C1* gene is highly expressed in hippocampal formation, its differential regulation by the different haplotypes might be related to the observed differences in cognitive function. Over-expression of *MAN2C1* leads to protein underglycolysation and up-regulation of the degradation of unfolded glycoproteins [19]. The attachment of glycans to some proteins is important for their correct folding and/or stability. N-glycans cover diverse biological functions in the nervous system, ranging from the essential to the modulation of development and neural transmission, which in turn can affect plasticity and memory formation [20]. Multiple glycosylation disorders with associated neurological symptoms and impaired cognitive ability have been reported [21]. An additional function of *MAN2C1*, independent of N-glycosylation, is apoptosis signalling and tumour growth [22–23]. Down-regulation of *MAN2C1* is linked to increments in apoptosis. The apoptotic action of the gene in the nervous system remains to be directly observed. Nevertheless, an indication of such function in the brain is given by previous findings showing that *MAN2C1* is over-expressed in patients with posttraumatic stress disorder [24–25], and that such psychopathology presents reduced apoptosis associated with defects in signal plasticity [26]. Therefore, the available data indicate that both over-expression and under-expression of *MAN2C1* can have a negative impact in brain function through different signalling pathways. A reading of our results consistent with *MAN2C1*’s literature further suggests that N2 could be linked to underglycolysation while N1b to mitochondrial-mediated apoptosis. More generally, our analyses indicate that the “where and how” genes are expressed in the brain is important for the interpretation of the associations between structural variations and cognitive phenotypes. Interestingly, we found a continuous growth of *MAN2C1* expression in brain during a life span, suggesting a more prominent role of glycosylation in development and apoptosis reduction at old age.

The convergence of two bioinformatics signals indicated that the haplotype structures could be supported by an inversion polymorphism between blocks C-D [11]. However, we could not find an inversion between CD and further experimental work is therefore needed. The small size of the single copy interval (<0.35 Mb) and the segmental duplications pose great difficulties to the experimental characterization by Next Generation data or cytogenetic methods such as FISH. Long reads such as those produce by PacBio could offer important insights. An unambiguous map between possible inversions at C-D and haplotype-genotypes is also challenged by the lack of tagging between the haplotype-genotypes in the proximal region between B-C and reciprocal reported inversions. Our data suggests, in particular, that the reported inversion at B-C may have occurred on a large haplotype background extending B-D and may not be causative of the haplotypes. This variable structure between individual chromosomes may add additional instability through meiotic mispairing and increased susceptibility to the reported recurrent germline rearrangements at 15q24 [4], as it has been shown in other genomic regions [27]. Given such complexity, more than one inversion configuration in size and extent is possible. That is, the orientation of the region the different segmental duplications may be polymorphic and its breakpoints extend to other segmental duplication blocks for different individuals. In addition, we cannot discard other mechanisms rather than inversions that could reduce recombination and sustain the haplotype-genotypes since migration from Africa such as structural variations within the segmental duplications or in the chromatin.

## Materials and Methods

### Detection and calling of large haplotype-genotypes

In this work, we used hg19 build as the reference, as it was the most common annotation over all datasets.

We used dense SNP data to detect large haplotype-genotypes in the chromosome band 15q24.1-15q24.3, prone to microdeletions and microduplications. It has been shown that the convergence of two different algorithms provide two distinct imprints of putative inversions on SNPs [11–12]. The first algorithm, inveRsion, is based on differences in LD between SNP blocks across inversion breakpoints [12]. This method allows one to search regions where unusually high linkage between distant points is observed. A positive signal between two points is given by the difference of Bayes Information Criterion (BIC), which, if greater than zero, indicates that the chromosomes of some individuals have higher SNP block linkage between the tested points than what would be expected under a null model. We first ran the inveRsion algorithm using 7 window sizes ranging from 0.4–1 Mb on the 73-79Mb (hg19) interval of the 15q24.1-15q24.3 genomic region, using the phased genotypes of 505 European individuals of the 1000 Genomes project (www.1000genomes.org). Genotypes with minor allele frequency of 0.01 were removed from the analysis.

The second algorithm, invClust [11], detects extended haplotype-genotypes that satisfy Hardy-Weinberg Equilibrium. It is based on the multivariate analysis of the SNPs within the interrogated region. The algorithm clusters individuals into haplotype-genotypes using the first two components of a multidimensional scaling (MDS) analysis of the SNPs in regions with positive signals given by inveRsion. We used a k-means method to identify the groups in the data produced by multiple allelic haplotypes. We then used data from 26 populations of the 1000 Genomes (http://www.1000genomes.org/) to study the population frequencies and heterozygosities as functions of distance from Ethiopia. Distances from Ethiopia were computed using likely migration paths around main water masses.

### Autism dataset

The Autism Genome Project (AGP) consortium represents an international effort collecting autism families for ongoing genetic studies. Cases were classified using the Autism Diagnostic Interview-Revised and Autism Diagnostic Observation Schedule instruments. We were granted permission to access genotype data of 2,563 trios obtained with the Human1M-Duo BeadChip (llumina) (ref: phs000267.v4.p2) (http://www.ncbi.nlm.nih.gov/gap). This dataset was used to define the Mendelian inheritance of inversion-haplotypes at 15q24.2 and to test their transmission disequilibrium in autism or autism spectrum disorder (ASD). We analysed 2,259 trios of European descent with ages between 4–18 years.

### General intelligence datasets

For association tests between IQ and inversion-related haplotypes, we used data from three independent cohorts of children and adolescents recruited from three different general populations: the INfancia y Medio Ambiente (INMA) Project, the Generation R (GenR) and the Saguenay Youth Study (SYS) (Figure A in S10 Fig and S4 File). All participants (or their parents in case of minors) gave written informed consent and local ethics committees approved each cohort study.The INMA sub-cohort studies were approved by the Clinical Research Ethics Committe (CEIC; Comité Ético de Investigación Clínica) of Barcelona (Reference No.: 2005/2106/1) the Generation R Study have been approved by the Medical Ethical Committee of the Erasmus Medical Center, Rotterdam.

#### INMA

INMA is a network of population-based birth cohorts across Spain [28] (http://www.proyectoinma.org/) established to study the impact of environmental pollutants on child development. We used a sub-sample of 909 genotyped children with a European ethnic origin, selected from 2042 individuals from the Menorca, Sabadell and Valencia cohorts. Children’s mothers were screened and recruited at the first-trimester ultrasound scan between 1997 and 2006. Genotyping was performed with HumanOmni1-Quad-BeadChip (Illumina). The McCarthy score of children’s abilities (MSCA) was administered at mean age of 4.8 years (SD = 0.45) in 1302 children, 906 of which were genotyped and had good quality testing, excluding incomplete administration, illness or tiredness. We selected the verbal and perceptive-performance scales of the MSCA.

#### GenR

This is a population-based cohort that at present covers fetal life to childhood of nearly 10,000 individuals born between 2002 and 2006 in Rotterdam, The Netherlands [29]. The data we used from this study comprised of genotypes from 1,236 European descendent children, obtained with Human610-Quad-BeadChip (Illumina). Intelligence measures were obtained at 6 years of age (SD = 0.4 years). Verbal IQ was assessed on 821 individuals using a subset of the Dutch battery [TaaltestvoorKinderen (TvK)], and nonverbal IQ was tested on 871 children using two subsets of the Dutch nonverbal intelligence test [Snijders-Oomen Niet-verbale intelligentie Test-Revisie (SON-R 2 ½ -7)] suited for children of 2.5–7 years of age. These two subsets tap into visuo-spatial abilities and abstract reasoning and their sum highly correlates with the full SON-R IQ battery.

#### SYS

We used data from the Saguenay Youth Study, established to study brain and cardio-metabolic health in 1,024 adolescents, 12 to 18 years of age (15±3.5), recruited and assessed in the Saguenay Lac-Saint-Jean region, Canada, between 2003 and 2012 [30]. Genotypes of 1,953 individuals, including parents, were obtained with Human610-Quad and HumanOmniExpress-BeadChips (Illumina), only SNPs present in both chips were analysed. We performed association tests on a subset of 1,011 adolescents who were tested with Wechsler Intelligence Scale for Children III (WISCIII). We used the verbal and performance components of the full test.

#### ORCADES

We also studied whether the genetic associations persisted in adults, using a cohort of 2,215 individuals with mean age of 54 years (± 15). The Orkney Complex Disease Study is a family-based study in the isolated Scottish archipelago of Orkney. Genetic diversity in this population is decreased compared to Mainland Scotland, consistent with the high levels of endogamy historically. Fasting blood samples were collected and over 500 health-related phenotypes and environmental exposures were measured in each individual. Cognitive verbal fluency was measured with the Mill Hill vocabulary scale.

While different IQ measures were obtained at different age groups (4, 6, between 12 and 18, and 54 years) in each of the cohorts, it is known that MSCA and WISC scores positively correlate for normal and cognitively impaired children [31]. On the other hand, the correlation between SON-R 2 ½ -7 and WISC-R is 0.8 [32]. All IQ measures were standardized to mean 100 and standard deviation of 15. As general IQ measures were not available in all cases, we analysed verbal and non-verbal IQ separately. Population characteristics (ethnicity, sex and age) and genome-wide principal components of the children in all cohorts were used as covariates (Figures B-D in S10 Fig).

### Expression datasets

We analysed gene expression levels in RNA from peripheral blood obtained in Estonian Gene Expression Cohort (EGCUT) (http://www.biobank.ee/). This cohort is composed of 1,074 randomly selected Estonian individuals (37+/-16.6 years; 50% females) from 53,000 subjects in the Estonian Genome Center Biobank, University of Tartu. Whole-Genome gene-expression levels were obtained by Illumina HT12v3 arrays according manufactures protocols. DNA was genotyped with Human370CNV array (Illumina). The final sample size with both genotype data and gene expression data was 882 individuals. We tested association between gene expression *in-cis* with haplotype-genotypes at N.

Data of the transcriptomic analysis of RNA from lymphoblastoid cell lines of CEU individuals were obtained from the European Bioinformatics Institute at EMBL (project E-MTAB-198) (http://www.ebi.ac.uk/arrayexpress/), to validate the findings on the previous dataset. For this analysis only parents were selected, removing grandparents NA12282 and NA12283 of family 1421. The final sample analysed here included 105 individuals.

Gene expression data in brain cortex, Myers et al. 2007 [33], was analysed for probes in *MAN2C1* and *SNUPN*. The data comprises 194 samples (with one removed after quality control) from the cerebral cortex of neurpathologically normal brains and was obtained with the Illunima HumanRefseq-8 Expression BeadChip. The individuals had self-defined ethnicity of European descent. For SNP genotyping, the Affymetrix GeneChip Human Mapping 500K Array Set was used. The expression and genotype data was downloaded from the website for The Laboratory of Functional Neurogenomics (http://labs.med.miami.edu/myers/).

For specific regions in the brain cortex, two gene expression data-sets were obtained from Gibbs et al 2010 [34] and BRAINEAC (http://www.braineac.org/), and analysed for *MAN2C1* and *SNUPN*, the gene that was found significant in all previous analyses. The first data-set (BRAINeQTL, dbGap accession number: phs000249.v1.p1) contains gene expression for 148 subjects in pons, cerebellum, temporal cortex and frontal cortex. The second data-set consists on expression and genotype data for 134 subjects in 10 different brain regions: White matter, cerebellum, medulla, hippocampus, putamen, substatia nigra, thalamus, occipital cortex, temporal cortex and frontal cortex. We downloaded data for 36 intragenic probes within *MAN2C1*.

### Data analyses

Recombination rates were downloaded from HapMap II (version 2011–01, http://hapmap.ncbi.nlm.nih.gov/downloads/recombination/) for chromosome 15. 10,000 random intervals of 3.15 Mb, corresponding to the A-D length, were extracted and their mean recombination rate computed. The average recombination rate in A-D was compared against this null distribution.

Phylogeny at N was reconstructed from the homozygous individuals N2, N1b and N1a from the 1000 genomes populations with the package phyclust (https://cran.r-project.org/web/packages/phyclust/index.html). We used the Hamming distance for the pair wise evolution distance between chromosomes.

Haplotype-genotypes at M, N, O and P were inferred by invClust as mentioned above and then treated as bi-allelic variants coded as (0,1,2) if the individuals had 0, 1 or 2 copies of M2, N2, O2 and P2. snpStats (https://www.bioconductor.org/packages/release/bioc/html/snpStats.html) was used to compute a wide range of statistics like linkage disequilibrium (r^2^) between the haplotypes and the SNPs in the region, Fixation index (FST), minor allele frequencies, Hardy-Weinberg Equilibrium and transmission disequilibrium tests for autistic children (TDT).

In our discovery sample, INMA, we fitted Gaussian regression models: IQ = hg + covariates, where hg are the haplotype genotypes corresponded to additive genetic models for M2, O2 and P2 and recessive for models for N2, N1a, N1b. Covariates were age of test administration, sex and first two PCA components to account for population stratification. The three haplotype structure at N was tested with recessive models for each haplotype to study its specific effect. Association tests between haplotype-genotypes and IQ were fitted with the linear function model (lm) of R.

Meta-analyses for associations between IQ measurements and the recessive models of N2, N1a N1b were performed with the rmeta package of R (https://cran.r-project.org/web/packages/rmeta/index.html). We fitted fixed model effects for cohorts, where the weights were the inverse of the variance estimates, to account for cohort differences and give more weight to larger studies or those with less variability. No significant heterogeneity between cohorts was observed for significant results which supports our choice of model.

To discover *in-cis* elements with haplotypes at N, we tested associations of gene expression in blood for the ECGUT data using regression models between the logarithm of normalized expression and additive models for N2, N1a and N1b. Results were deemed significant for p-values < 4 10^−3^ to correct for the test of 11 genes in the region. Validation on the un-demented individual samples was tested with similar regression models on probes in *MAN2C1* and *SNUPN*. We recover recessive models of N2, N1a and N1b to test their association between the logarithms of normalized gene expression levels in different brain regions and, hence, study the specific effect of each haplotype, likewise the IQ associations.

## Supporting Information

S1 Fig Variance of haplotype frequency explained by the distance from Ethiopia (R^2^) for M2, N2, O2 and P2, compared with those from 10,000 random SNPs in the genome that matched the mean African frequencies of each haplotype. (TIF)

S2 Fig Pair wise Fst as function of geographical distance between populations. (TIF)

S3 Fig Synteny analysis between the reference genome assembly NCBI 36/hg18 and the rat genome assembly RGSC 5.0/rn5. (TIF)

S4 Fig Synteny analysis between *Mus musculus* (MM), *Homo sapiens* (HS), *Pongo pygmaeus abelii* (PPY) and *Macaca mulatta* (MMU). In HS, tag SNPs showed that BAC libraries RP11, CTD and CTA belong to N2, N1a and N1b haplotypes. Black squares show gaps in BAC libraries.
(TIF)

S5 Fig FISH between blocks CD to determine possible inversion at N. (TIF)

S6 Fig FISH to determine possible inversion at B-D in haplotypes. The experiments discard possible inversions for haplotypes N2, N1a and N1b between blocks B-D.
(TIF)

S7 Fig Haplotype-genotyping at N for A) INMA, B) GenR and C) SYS.
(TIF)

S8 Fig Meta-analysis of verbal IQ, including ORCHADES cohort. P-value for the association with N2 is increased.
(TIF)

S9 Fig *MAN2C1* expression in human brain as a function of age. NCX: Cortex, STR: Striatum, Hip: Hippocampus, MD: Medula, AMY: Amygdala, CBC: Cerebellum.
(TIF)

S10 Fig Study characteristics and PCAs. A) Characteristics of children cohorts used in the study. First to genome-wide principal component analysis for B) INMA, C) GenR, D) SYS and E) AGP.
(TIF)

S1 File N2, N1b and N1a genotypes of 60 trios from CEU and YRI populations, and 2,259 trios from AGP. (XLS)

S2 File SNPs in high linkage with haplotypes N2, N1a and N1b for CEU YRI and CHP+JPT of HapMap. (XLS)

S3 File SNPs in high LD with N2, N1a and N1b and BAC coverage of flanking sequences. (XLSX)

S4 File IQ phenotypes and inversion genotypes at 15q24 for INMA, SYS and ORCADES studies. GenR data is available upon request.
(XLS)

S1 Table Frequencies and Hardy-Weinberg Equilibrium p-values of M2, N2, O2 and P2 for the 26 populations of the 1000 Genomes project. (TIF)

S2 Table Association between haplotypes at 15q24.2 and local gene expression for EGCUT and CEU. (TIF)

## References

[1] Kidd JM, Cooper GM, Donahue WF, Hayden HS, Sampas N, Graves T, et al Mapping and sequencing of structural variation from eight human genomes. Nature. 2008; 453:56–64. 10.1038/nature06862 18451855 PMC2424287

[2] Antonacci F, Kidd JM, Marques-Bonet T, Ventura M, Siswara P, Jiang Z, et al Characterization of six human disease-associated inversion polymorphisms. Hum Mol Genet 2009;18: 2555–2566. 10.1093/hmg/ddp187 19383631 PMC2701327

[3] Magoulas PL, El-Hattab AW. Chromosome 15q24 microdeletion syndrome. Orphanet J Rare Dis. 2012;7: 2 10.1186/1750-1172-7-2 22216833 PMC3275445

[4] Mefford HC, Rosenfeld JA, Shur N, Slavotinek AM, Cox VA, Hennekam RC, et al Further clinical and molecular delineation of the 15q24 microdeletion syndrome. J Med Genet. 2012; 49: 110–118. 10.1136/jmedgenet-2011-100499 22180641 PMC3261729

[5] Roetzer KM, Schwarzbraun T, Obenauf AC, Hauser E, Speicher MR. Further evidence for the pathogenicity of 15q24 microduplications distal to the minimal critical regions. Am J Med Genet Part A. 2010;152: 3173–3178. 10.1002/ajmg.a.33750 21108404

[6] Sharp AJ, Selzer RR, Veltman JA, Gimelli S, Striano P, Coppola A, et al Characterization of a recurrent 15q24 microdeletion syndrome. Hum Mol Genet. 2007; 16: 567–572. 17360722 10.1093/hmg/ddm016

[7] McInnes LA, Nakamine A, Pilorge M, Brandt T, Jiménez González P, Fallas M, et al A large-scale survey of the novel 15q24 microdeletion syndrome in autism spectrum disorders identifies an atypical deletion that narrows the critical region. Mol Autism. 2010; 19:5–5. 10.1186/2040-2392-1-5 PMC2907565 20678247

[8] González JR, Cáceres A, Esko T, Cuscó I, Puig M, Esnaola M, et al A common 16p11. 2 inversion underlies the joint susceptibility to asthma and obesity. Am J Hum Genet. 2014; 94: 361–372. 10.1016/j.ajhg.2014.01.015 24560518 PMC3951940

[9] Salm MP, Horswell SD, Hutchison CE, Speedy HE, Yang X, Liang L, et al The origin, global distribution, and functional impact of the human 8p23 inversion polymorphism. Genome Res. 2012; 22: 1144–1153. 10.1101/gr.126037.111 22399572 PMC3371712

[10] Steinberg KM, Antonacci F, Sudmant PH, Kidd JM, Campbell CD, Vives L, et al Structural diversity and African origin of the 17q21.31 inversion polymorphism. Nat Genet. 2012;44: 872–880. 10.1038/ng.2335 22751100 PMC3408829

[11] Cáceres A, González JR. Following the footsteps of inversions on SNP data: From detection to association tests. Nucleic Acids Res. 2015;43: e53–e53. 10.1093/nar/gkv073 25672393 PMC4417146

[12] Cáceres A, Sindi SS, Raphael BJ, Cáceres M, González JR. Identification of polymorphic inversions from genotypes. BMC bioinformatics 2012;13: 28 10.1186/1471-2105-13-28 22321652 PMC3296650

[13] Voight BF, Kudaravalli S, Wen X, Pritchard JK. A map of recent positive selection in the human genome. PLoS Biol. 2006;4: 446–446. 10.1371/journal.pbio.0040072 PMC1382018 16494531

[14] Paterson T, Law A, Arkmap: integrating genomic maps across species and data sources. BMC bioinformatics 2013; 14:246 10.1186/1471-2105-14-246 23941167 PMC3751345

[15] Bhutta AT, Cleves MA, Casey PH, Cradock MM, Anand KJS. Cognitive and behavioral outcomes of school-aged children who were born preterm: a meta-analysis. Jama. 2002;288: 728–737. 12169077 10.1001/jama.288.6.728

[16] Benyamin B, Pourcain B, Davis O, Davies G, Hansell N, Brion MJ, et al Childhood intelligence is heritable, highly polygenic and associated with fnbp1l. Mol Psychiatr. 2013;19: 253–258. 10.1038/mp.2012.184 PMC3935975 23358156

[17] Narayanan U, Ospina JK, Frey MR, Hebert MD, Matera AG. Smn, the spinal muscular atrophy protein, forms a pre-import snrnp complex with snurportin1 and importin β. Hum Mol Genet. 2002;11: 1785–1795. 12095920 10.1093/hmg/11.15.1785 PMC1630493

[18] Zielinska DF, Gnad F, Wiśniewski JR, Mann M. Precision mapping of an in vivo n-glycoproteome reveals rigid topological and sequence constraints. Cell 2010;141: 897–907. 10.1016/j.cell.2010.04.012 20510933

[19] Bernon C, Carré Y, Kuokkanen E, Slomianny MC, Mir AM, Krzewinski F, et al Overexpression of MAN2C1 leads to protein underglycosylation and upregulation of endoplasmic reticulum-associated degradation pathway. Glycobiology 2011;21: 363–375. 10.1093/glycob/cwq169 20978011

[20] Scott H, Panin VM. The role of protein N-glycosylation in neural transmission. Glycobiology 2014;24: 407–417. 10.1093/glycob/cwu015 24643084 PMC3976283

[21] Freeze HH, Eklund EA, Ng BG, Patterson MC. Neurology of inherited glycosylation disorders. Lancet Neurol. 2012;11: 453–466. 10.1016/S1474-4422(12)70040-6 22516080 PMC3625645

[22] Xiang Z, Jiang D, Liu Y, Zhang L, Zhu L. hMAN2C1 transgene promotes tumor progress in mice. Transgenic Res. 2010;19: 67–75. 10.1007/s11248-009-9299-3 19572206

[23] Wang L, Suzuki T. Dual functions for cytosolic α-mannosidase (MAN2C1) its down-regulation causes mitochondria-dependent apoptosis independently of its α-mannosidase activity. J Biol Chem 2013;288: 11887–11896. 10.1074/jbc.M112.425702 23486476 PMC3636876

[24] Yehuda R, Cai G, Golier JA, Sarapas C, Galea S, Ising M, et al Gene expression patterns associated with posttraumatic stress disorder following exposure to the world trade center attacks. Biol Psychiat. 2009;66: 708–711. 10.1016/j.biopsych.2009.02.034 19393990

[25] Uddin M, Galea S, Chang SC, Aiello AE, Wildman DE, de los Santos R, et al Gene expression and methylation signatures of MAN2C1 are associated with ptsd. Dis Markers. 2011;30: 111–121. 10.3233/DMA-2011-0750 21508515 PMC3188659

[26] Mkrtchyan G, Boyadzhyan A, Avetyan D, Sukiasyan S. Involvement of anomalous apoptosis in impairments to synaptic plasticity in post-traumatic stress disorder. Neurosci Behav Physiol. 2014;44: 442–446.

[27] Cuscó I, Corominas R, Bayés M, Flores R, Rivera-Brugués N, Campuzano V, et al Copy number variation at the 7q11. 23 segmental duplications is a susceptibility factor for the williams-beuren syndrome deletion. Genome Res. 2008;18: 683–694. 10.1101/gr.073197.107 18292220 PMC2336808

[28] Guxens M, Ballester F, Espada M, Fernández MF, Grimalt JO, Ibarluzea J, et al Cohort profile: the inma—infancia y medio ambiente—(environment and childhood) project. Int J Epidemiol. 2012;41: 930–940. 21471022 10.1093/ije/dyr054

[29] Jaddoe VW, van Duijn CM, van der Heijden AJ, Mackenbach JP, Moll HA, Steegers EA, et al The generation r study: design and cohort update 2010; Eur J Epidemiol. 2010; 25: 823–841. 10.1007/s10654-010-9516-7 20967563 PMC2991548

[30] Pausova Z, Paus T, Abrahamowicz M, Almerigi J, Arbour N, Bernard M, et al Genes, maternal smoking, and the offspring brain and body during adolescence: design of the Saguenay youth study. Hum Brain Mapp 2007;28: 502–518. 17469173 10.1002/hbm.20402 PMC6174527

[31] Naglieri JA. Comparison of McCarthy general cognitive index and WISC-R IQ for educable mentally retarded, learning disabled and normal children, Psychol. Rep. 1980;47: 591–596. 7454913 10.2466/pr0.1980.47.2.591

[32] Tellegen PJ, Laros JA. The Snijders-Oomen nonverbal intelligence tests: general intelligence tests or tests for learning potential In: Hamers JHM, Sijtsma K, Ruijssenaars AJJM, editors. Learning Potential Assessment, Theoretical, Methodological and Practical Issues. Amsterdam: Swets & Zeitlinger; 1993 pp 267–283.

[33] Myers AJ, Gibbs JR, Webster JA, Rohrer K, Zhao A, Marlowe L, et al A survey of genetic human cortical gene expression. Nat. Genet. 2007;39: 1494–1499. 17982457 10.1038/ng.2007.16

[34] Gibbs JR, van der Brug MP, Hernandez DG, Traynor BJ, Nalls MA, Lai SL, et al Abundant quantitative trait loci exist for DNA methylation and gene expression in human brain. PLoS Genet. 2010;13: e1000952. 10.1371/journal.pgen.1000952 PMC2869317 20485568

