# Supplementary figures and images for: Ancient Haplotypes at the 15q24.2 Microdeletion Region Are Linked to Brain Expression of MAN2C1 and Children's Intelligence
Source: PLoS One. 2016 Jun 29;11(6):e0157739. doi: 10.1371/journal.pone.0157739 (PMC4927142; doi:10.1371/journal.pone.0157739)

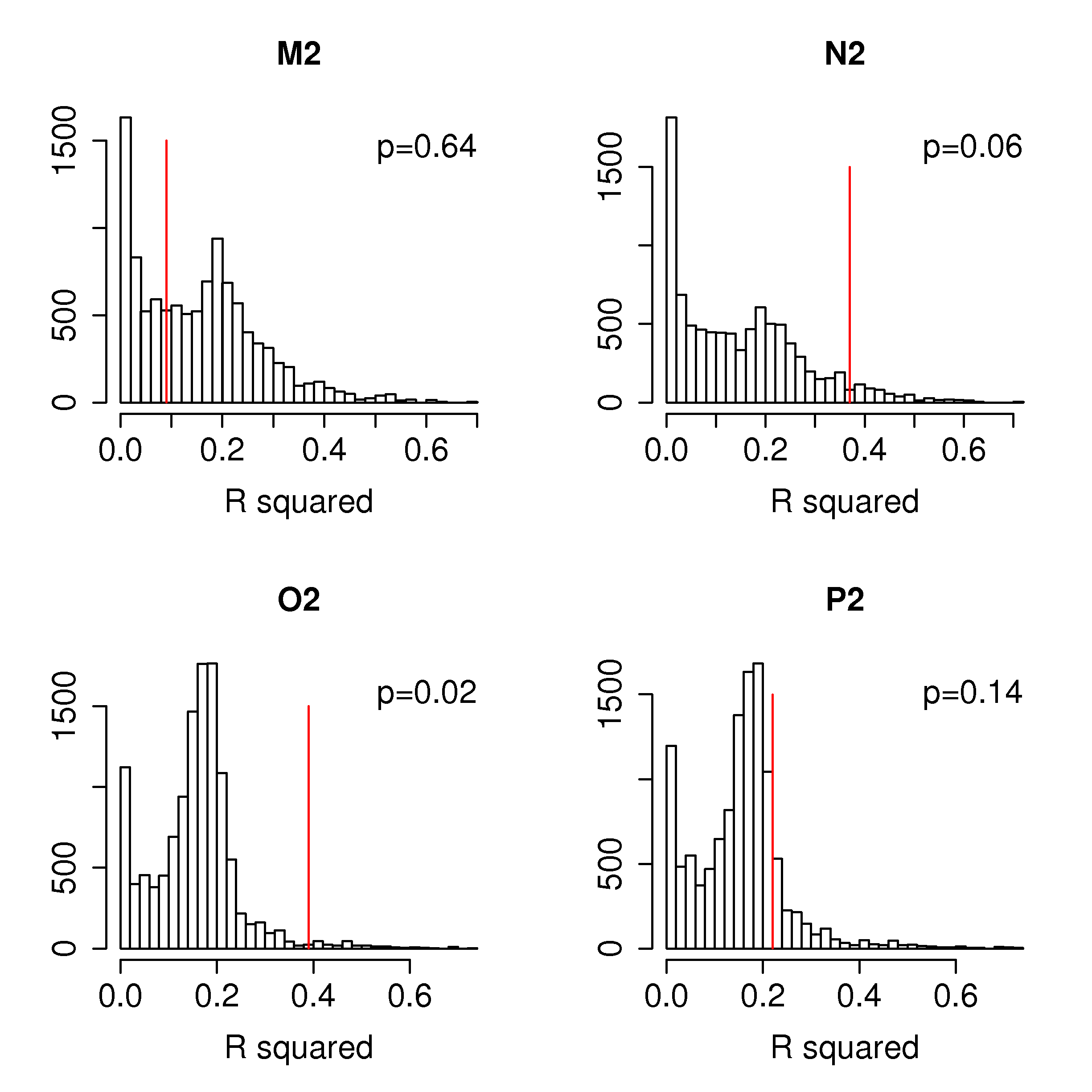

Supplement: S1 Fig — (TIF) [file pone.0157739.s001.tif]

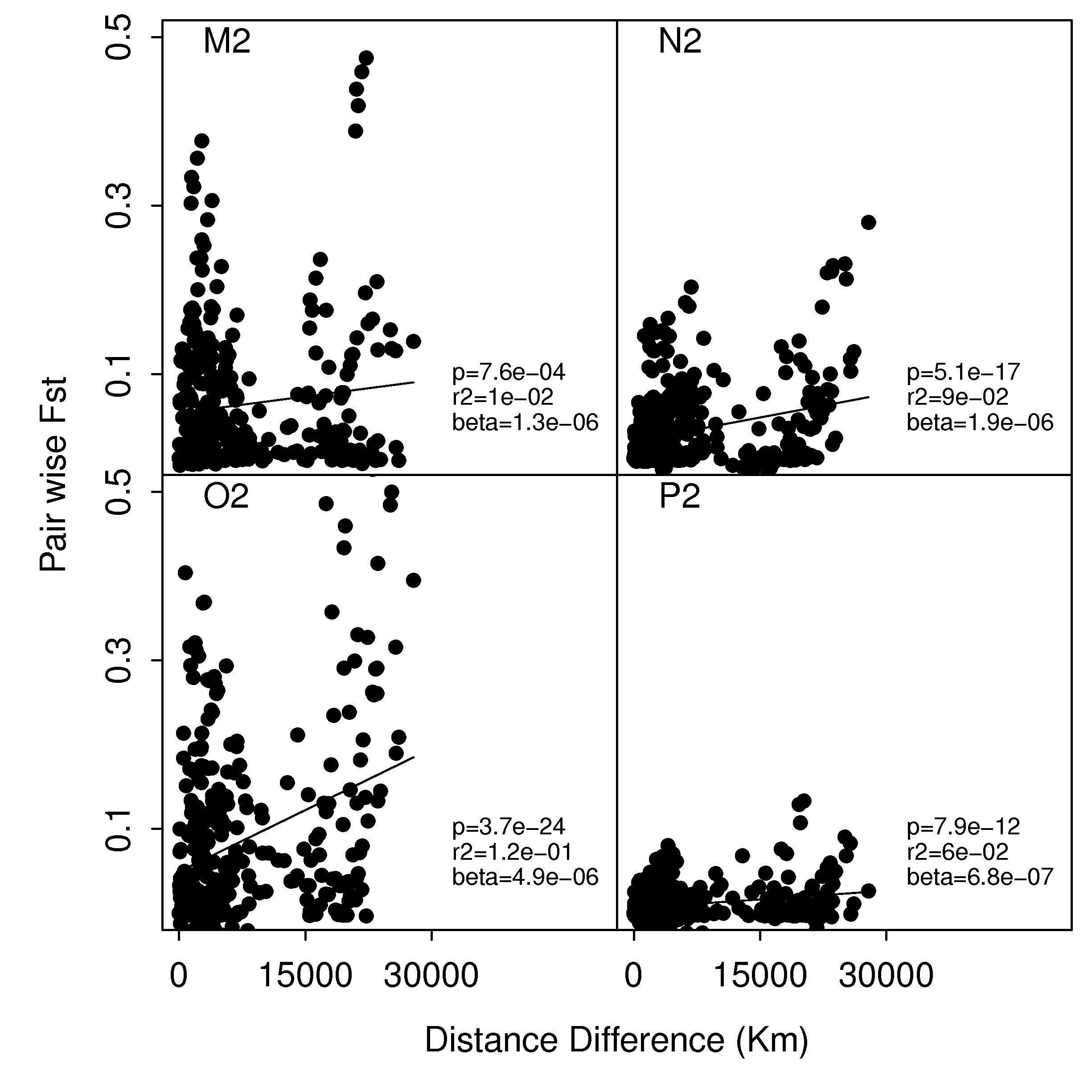

Supplement: S2 Fig — (TIF) [file pone.0157739.s002.tif]

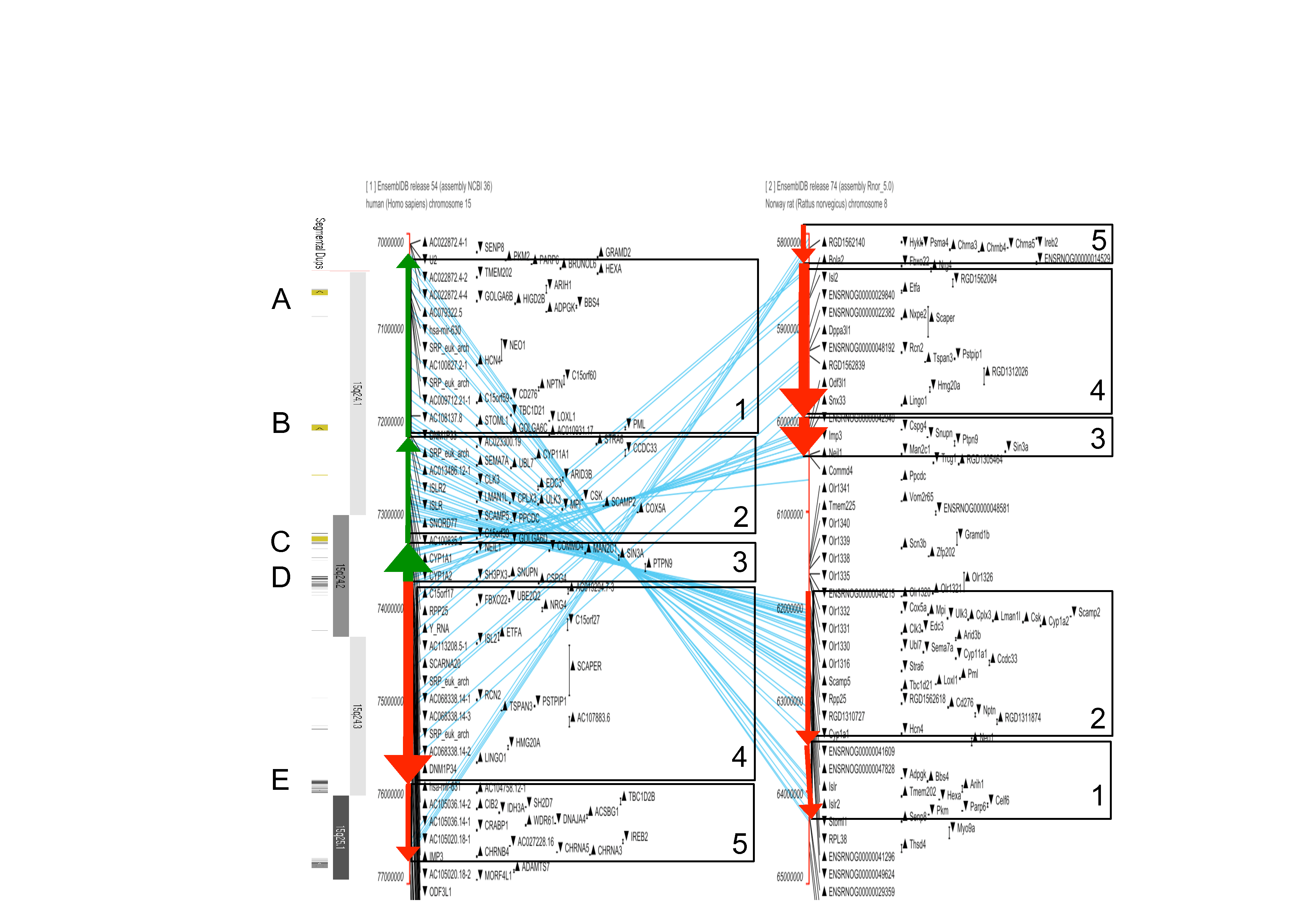

Supplement: S3 Fig — (TIF) [file pone.0157739.s003.tif]

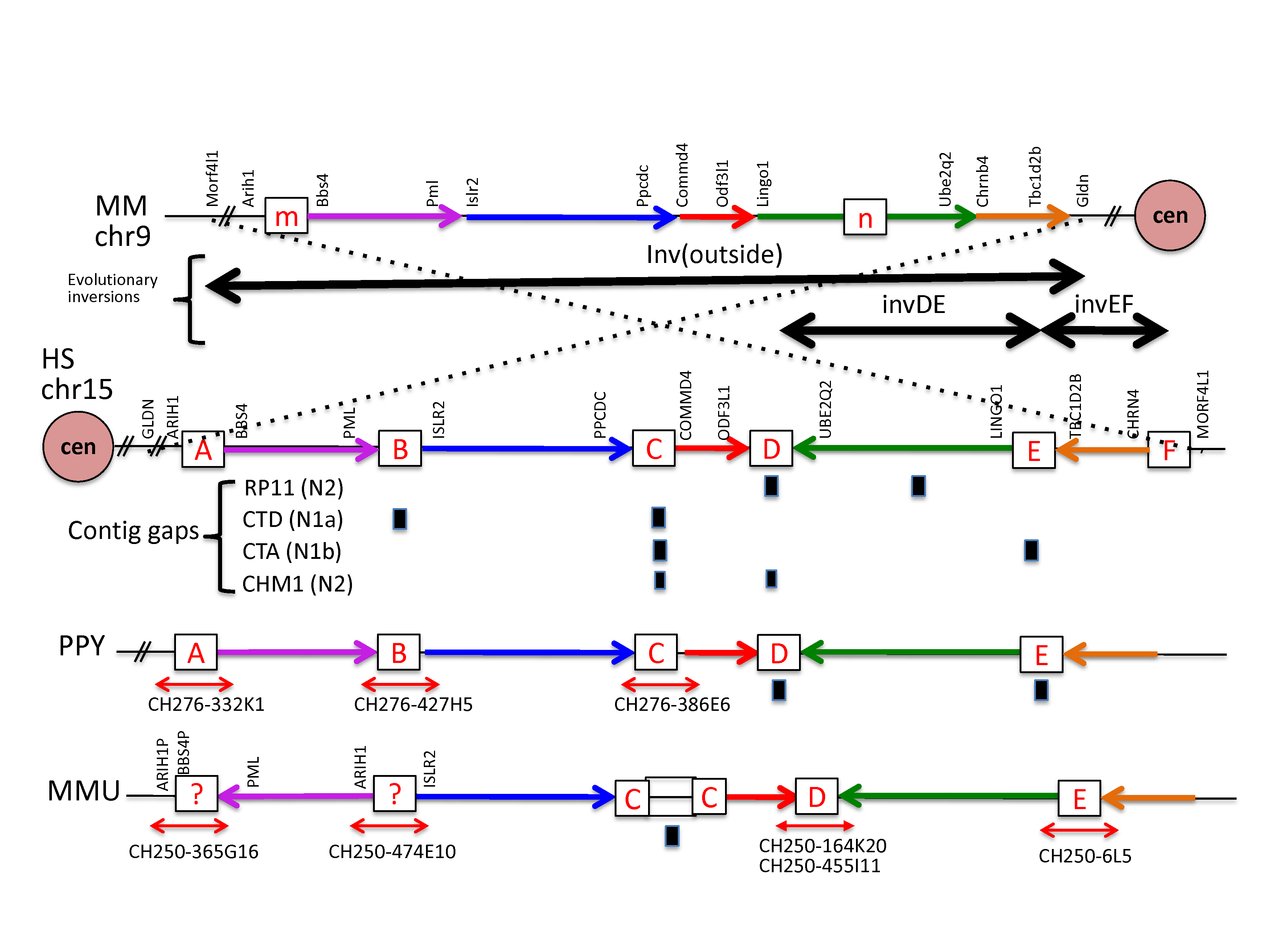

Supplement: S4 Fig — In HS, tag SNPs showed that BAC libraries RP11, CTD and CTA belong to N2, N1a and N1b haplotypes. Black squares show gaps in BAC libraries. (TIF) [file pone.0157739.s004.tif]

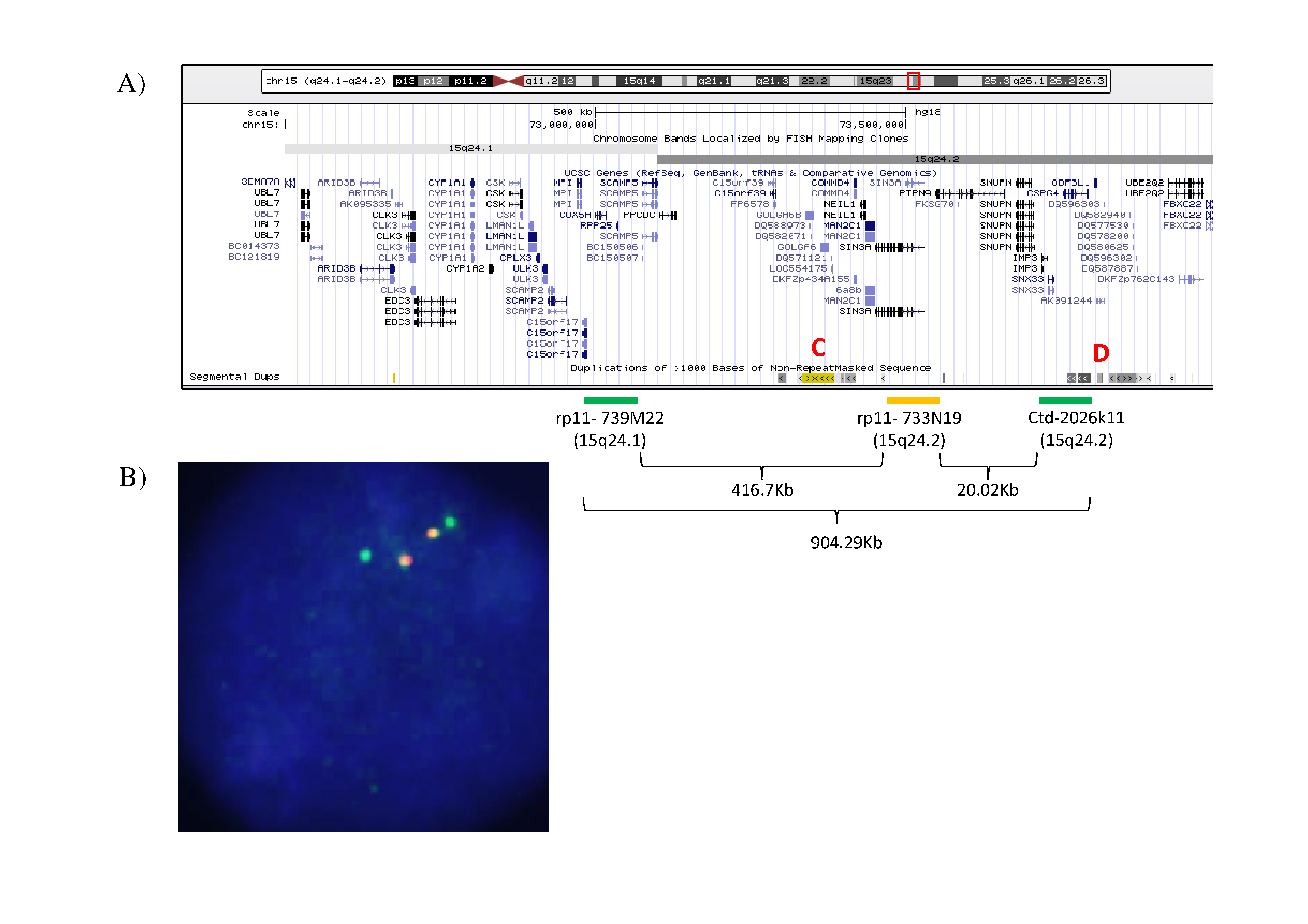

Supplement: S5 Fig — (TIF) [file pone.0157739.s005.tif]

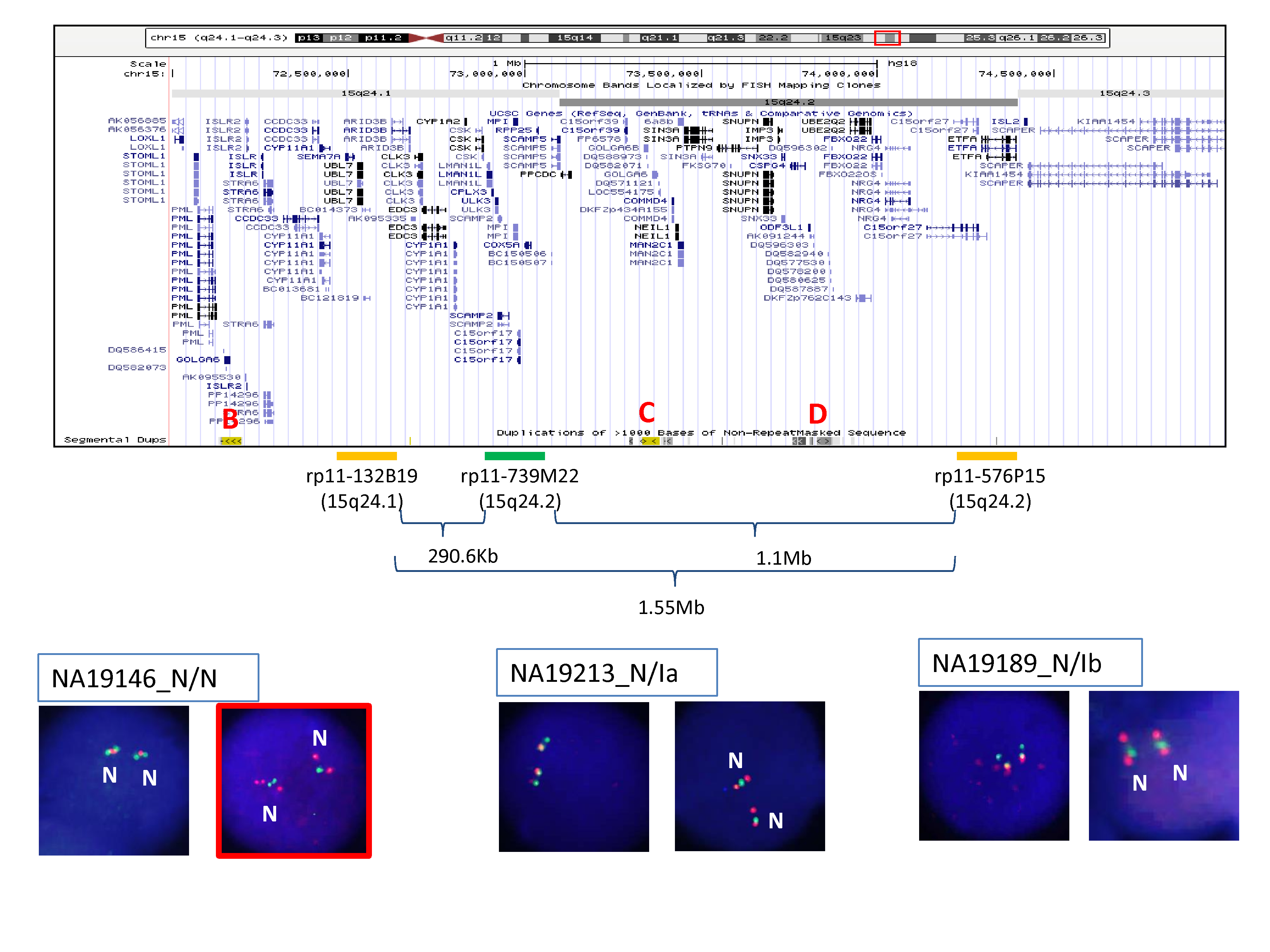

Supplement: S6 Fig — The experiments discard possible inversions for haplotypes N2, N1a and N1b between blocks B-D. (TIF) [file pone.0157739.s006.tif]

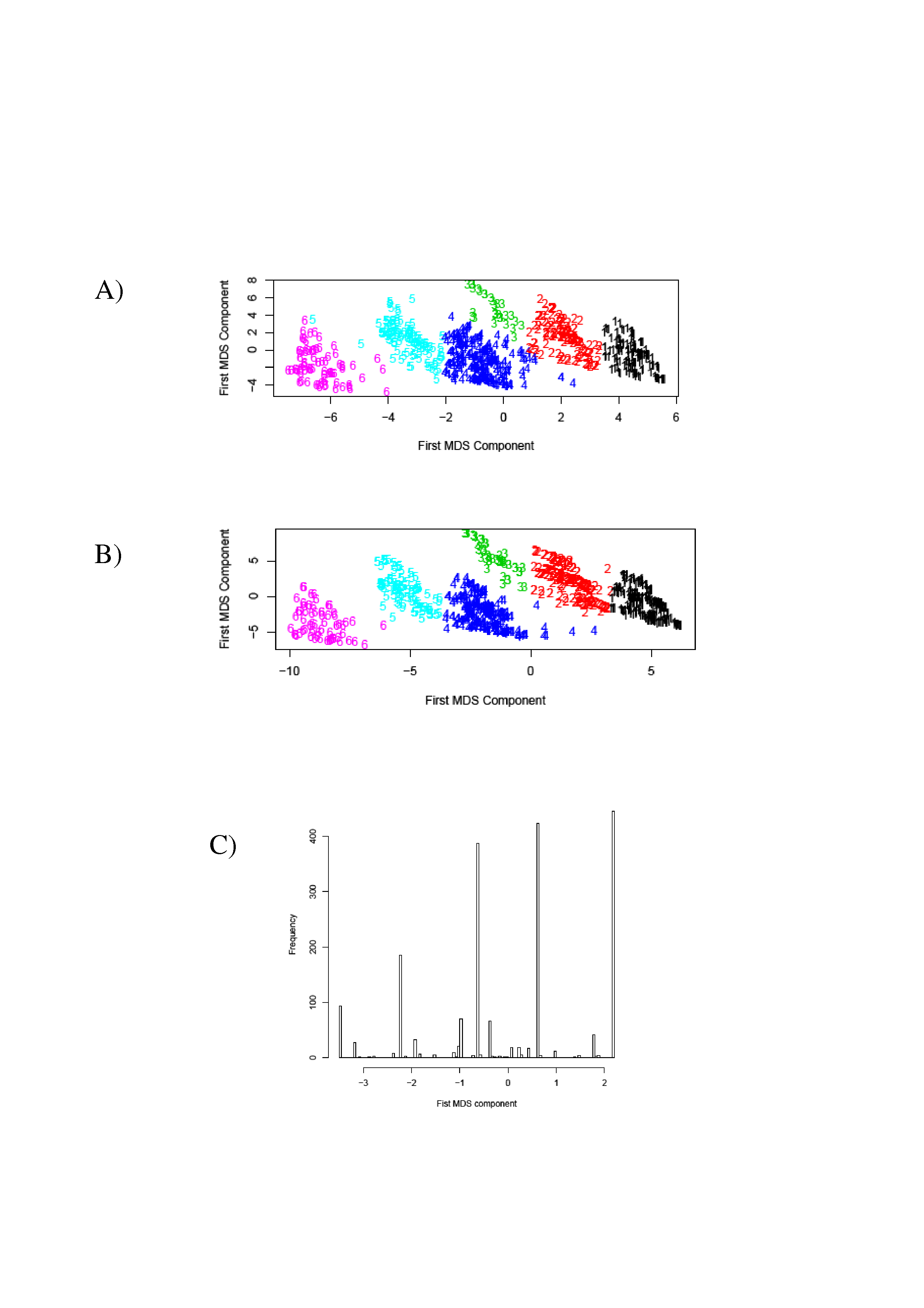

Supplement: S7 Fig — Haplotype-genotyping at N for A) INMA, B) GenR and C) SYS. (TIF) [file pone.0157739.s007.tif]

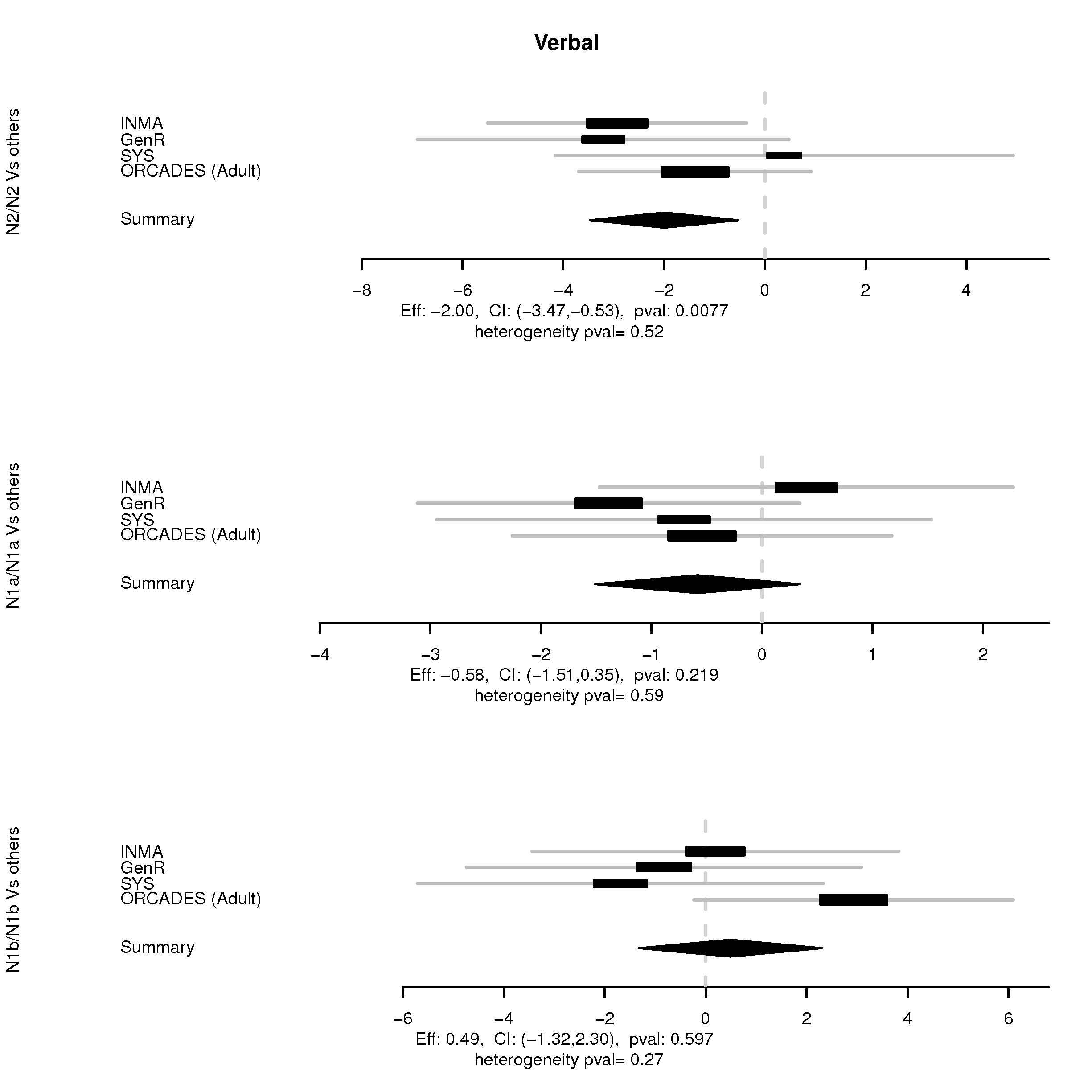

Supplement: S8 Fig — P-value for the association with N2 is increased. (TIF) [file pone.0157739.s008.tif]

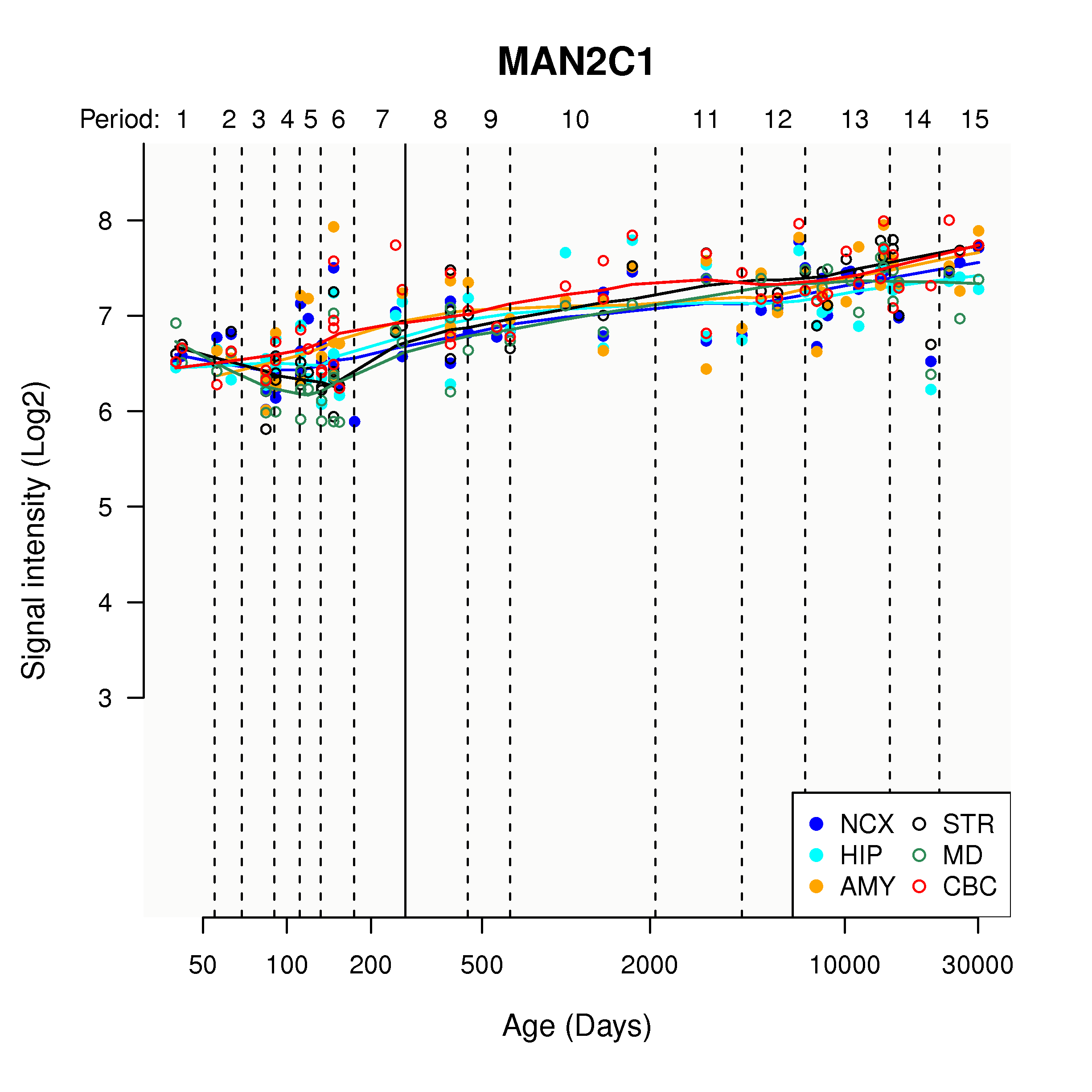

Supplement: S9 Fig — NCX: Cortex, STR: Striatum, Hip: Hippocampus, MD: Medula, AMY: Amygdala, CBC: Cerebellum. (TIF) [file pone.0157739.s009.tif]

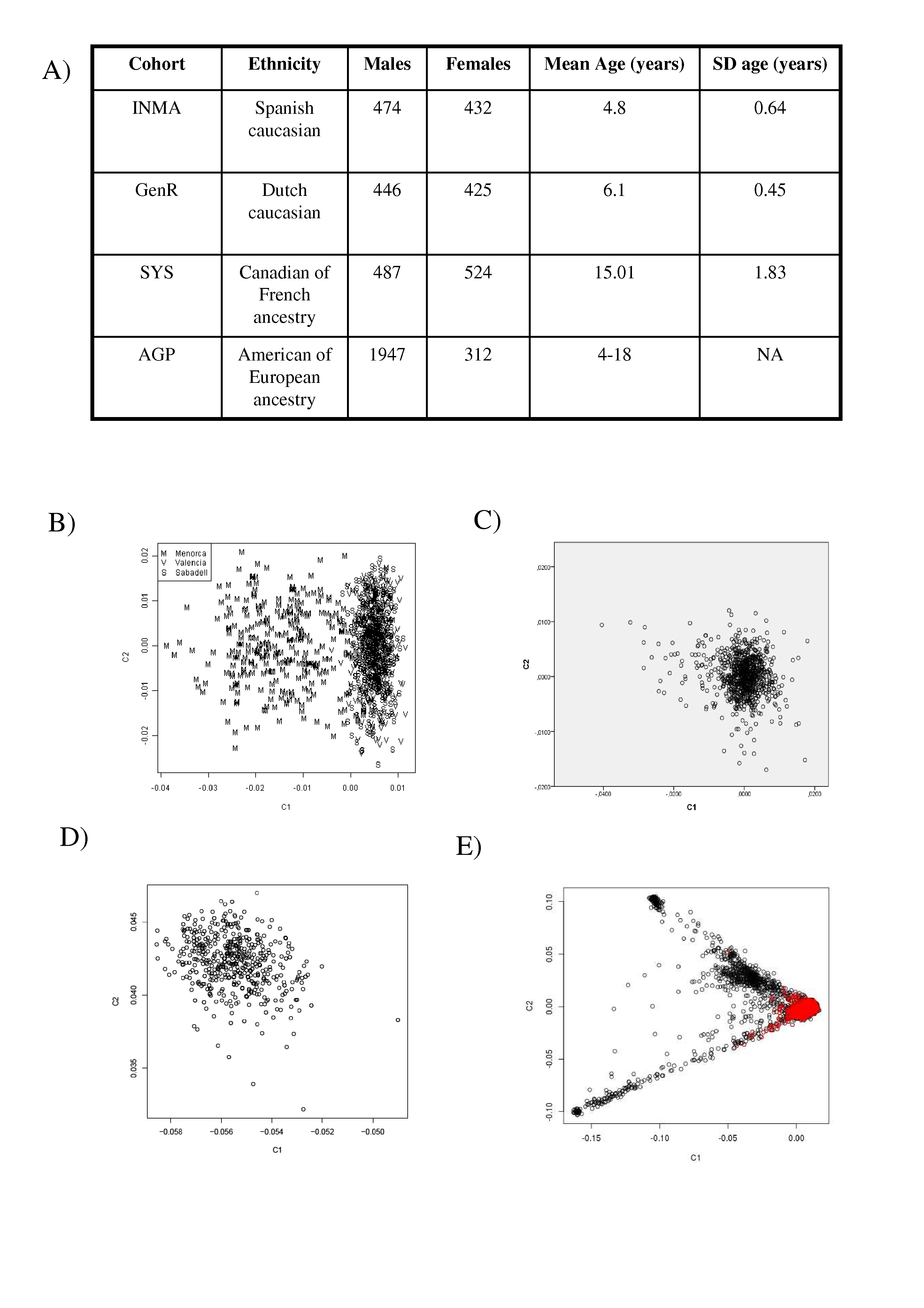

Supplement: S10 Fig — A) Characteristics of children cohorts used in the study. First to genome-wide principal component analysis for B) INMA, C) GenR, D) SYS and E) AGP. (TIF) [file pone.0157739.s010.tif]

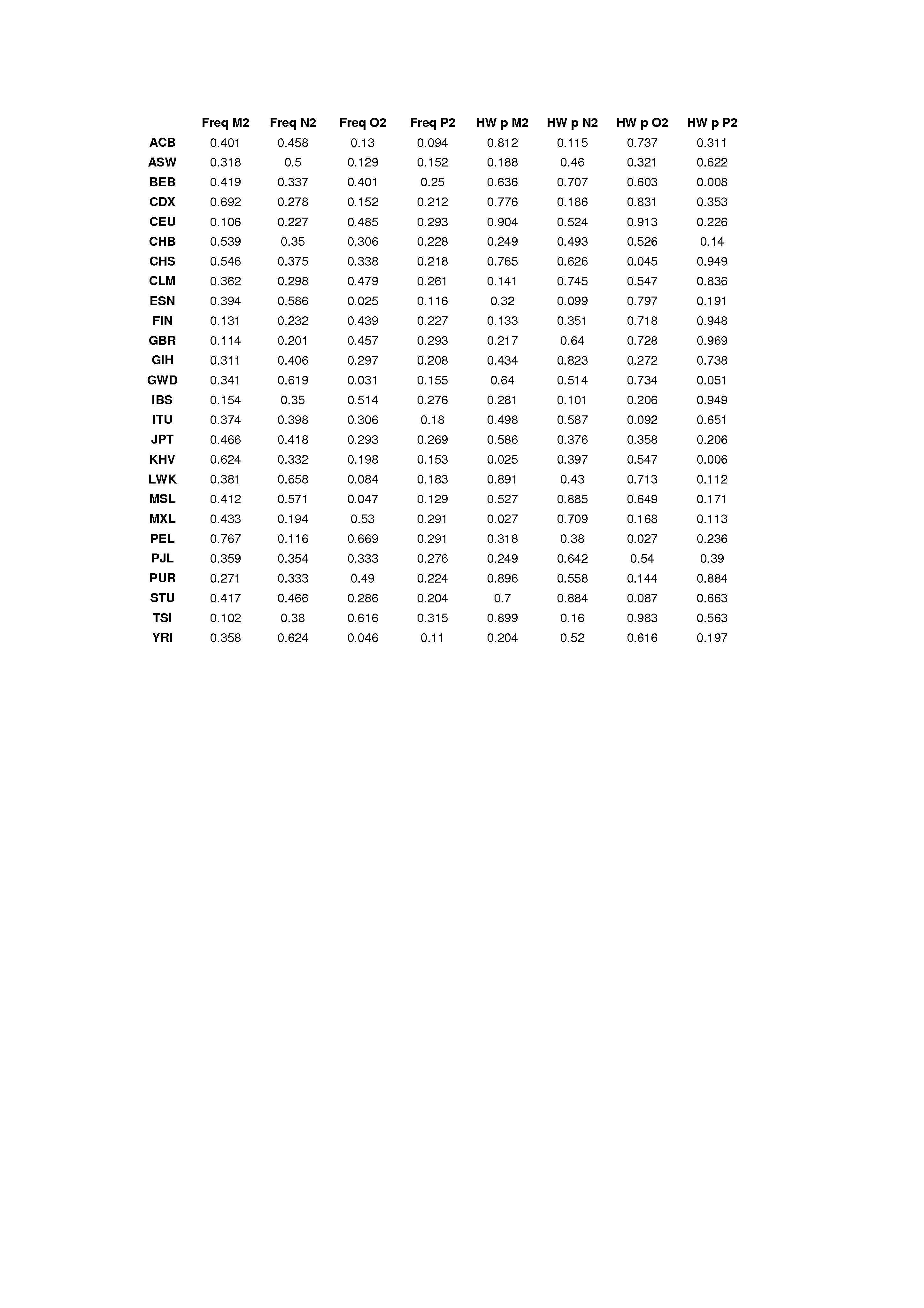

Supplement: S1 Table — (TIF) [file pone.0157739.s015.tif]

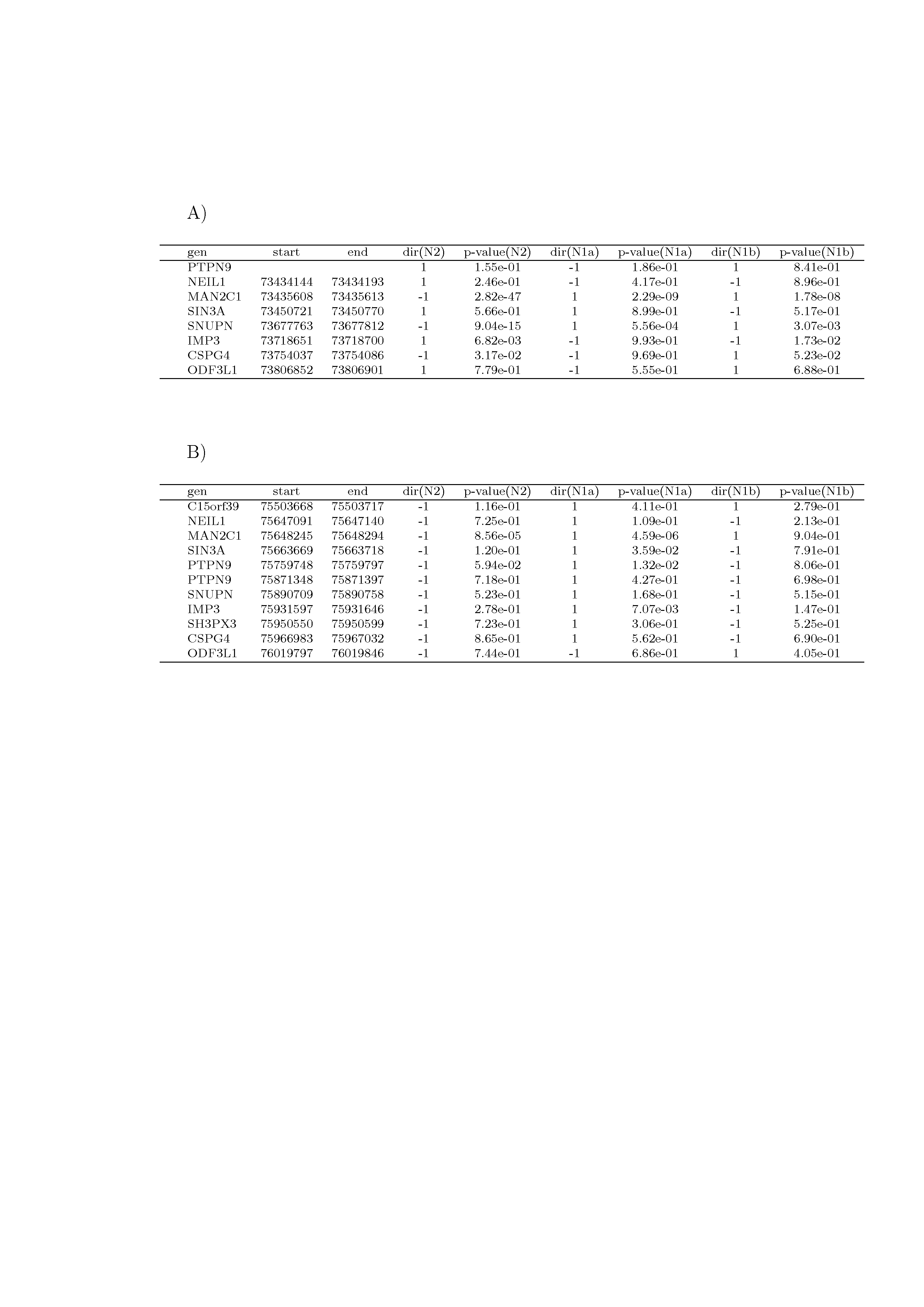

Supplement: S2 Table — (TIF) [file pone.0157739.s016.tif]
